# Mixed-mobility supported lipid bilayers uncover the role of immobilized ICAM1 on T cell activation and immune synapse organization

**DOI:** 10.1073/pnas.2530126123

**Published:** 2026-03-10

**Authors:** Alexander Leithner, Audun Kvalvaag, Tanmay Mitra, Salvatore Valvo, Hannah Dada, Ewoud Compeer, Michael I. Barton, Sofia Bustamante Eguiguren, Edward Jenkins, Christoffer Lagerholm, Omer Dushek, Michael L. Dustin

**Affiliations:** ^a^The Kennedy Institute of Rheumatology, Nuffield Department of Orthopaedics, Rheumatology and Musculoskeletal Sciences (NDORMS), University of Oxford, Oxford OX3 7FY, United Kingdom; ^b^Department of Molecular Cell Biology, Institute for Cancer Research, Oslo University Hospital, Oslo 0379, Norway; ^c^Chinese Academy of Medical Science Oxford Institute, Nuffield Department of Medicine, University of Oxford, Oxford OX3 7BN, United Kingdom; ^d^Sir William Dunn School of Pathology, University of Oxford, Oxford OX1 3RE, United Kingdom

**Keywords:** immunological synapse, ICAM1, mobility, cytotoxicity, force

## Abstract

The immunological synapse (IS) integrates antigen recognition and adhesion to control T cell activation and effector functions. Reductionist systems have been instrumental in dissecting IS organization, but conventional systems constrain all ligands to be either mobile or immobile, unlike antigen-presenting cells where intercellular adhesion molecule 1 (ICAM1) is cytoskeletally anchored while T cell receptor (TCR) ligands remain mobile. Here, we establish mixed-mobility supported lipid bilayers (SLBs) that simultaneously present mobile TCR agonists and immobile ICAM1. Selective immobilization of ICAM1 disrupts centripetal F-actin flow, prevents centralization of TCR microclusters and shifts signaling to peripheral microclusters. This attenuates TCR downregulation through ectocytosis while maintaining recycling, and enhances integrin mechanotransduction, reflected in increased phosphorylation of Focal Adhesion Kinase, Paxillin, and the stretch-sensitive adaptor CasL. Functionally, immobilized ICAM1 augments T cell activation, degranulation, Perforin release, and cytotoxicity. Importantly, these findings were recapitulated in a cell–cell system engineered to express either full-length, cytoskeleton-anchored ICAM1 or a truncated form lacking cytoskeletal association, with full-length ICAM1 consistently promoting stronger effector responses. These findings identify ligand mobility as a key biophysical parameter that shapes IS organization and T cell effector responses and establish mixed-mobility SLBs as a powerful tool for probing receptor mechanics in immunity.

## Significance

This research explores how the mobility of a key immune ligand, intercellular adhesion molecule 1 (ICAM1), influences T cell activation. T cells have a receptor that specifically binds to ICAM1, triggering events that contribute to T cell activation. By creating a system where ICAM1 is selectively immobilized or mobile on a surface, this study shows that when ICAM1 is anchored, T cells become more activated and increase their ability to kill target cells. These findings reveal that the physical anchoring of molecules in immune interactions can significantly impact how T cells respond. This could help design better strategies for enhancing immune responses, such as in cancer therapies, by modulating how immune receptors and ligands are anchored during interactions.

The immunological synapse (IS) is a highly organized cell–cell interface that governs critical T cell functions, including antigen recognition, signal integration, and effector responses (1, 2). Mechanistic insights into IS formation have largely emerged from a number of reductionist in vitro systems. Initial seminal studies with cell lines serving as model antigen-presenting cells (APCs) first demonstrated formation of the central supramolecular activation cluster (cSMAC), enriched for T cell receptor (TCR) and protein kinase C-θ, surrounded by the peripheral SMAC enriched for integrin lymphocyte function-associated antigen 1 (LFA-1), cytoskeletal adapter talin, and F-actin (3, 4). Glass-supported lipid bilayers (SLBs), presenting laterally mobile peptide major histocompatibility complex (pMHC) or TCR agonist and Intercellular adhesion molecule 1 (ICAM1) generated similar IS dynamics and organization, while enabling quantitative, high-resolution microscopy (5–8). In parallel, glass substrates coated with immobile TCR agonists (9) provided insights into microclusters, the basic TCR signaling unit, which were also glimpsed with model APCs (4) and extensively studied in SLBs (10).

It has become clear that the originally employed model APCs lack active cytoskeletal processes that increase T cell activation by dendritic cells (DCs), which form multifocal instead of monofocal synapses (11–17). Furthermore, cytoskeletal anchorage controls lateral mobility of ICAM1 on mature DCs leading to enhanced T cell activation, while MHC molecules remain highly mobile (18). SLBs presenting mobile TCR agonists interrupted by micrometer-scale clusters of immobile vascular cell adhesion molecule 1 (VCAM-1), another integrin ligand, suppressed centralizing F-actin flow, which might account for failure of monofocal synapse formation (19). However, this patterned, mixed-mobility substrate does not allow isolation of lateral mobility from other parameters, such as ligand clustering, that are likely to be important for T cell responses.

To directly test how ICAM1 anchorage influences T cell IS formation, activation, and degranulation, we developed a spatially integrated mixed-mobility SLB platform. In this system, ICAM1 is rendered immobile at the nanometer scale without forming discrete micron-scale islands, ensuring continuous spatial integration with mobile ligands. This enables independent control of ICAM1 lateral mobility while preserving the lateral mobility of TCR ligands, allowing us to directly examine TCR microcluster formation, centripetal transport, and force-dependent signaling dynamics under defined integrin anchorage conditions. Using this system, we demonstrate that selective ICAM1 immobilization profoundly influences multiple aspects of IS formation, including T cell spreading dynamics, F-actin cytoskeleton organization, TCR microcluster transport, TCR recycling, and integrin activation. Importantly, we show that ICAM1 immobilization enhances T cell degranulation, findings further supported by increased cytotoxicity in cell–cell killing assays.

Together, these results highlight the importance of mobility regulation as a key biophysical parameter that shapes T cell responses and emphasize its relevance for both mechanistic studies and the design of future immunotherapies.

## Results

**Fig. 1.**
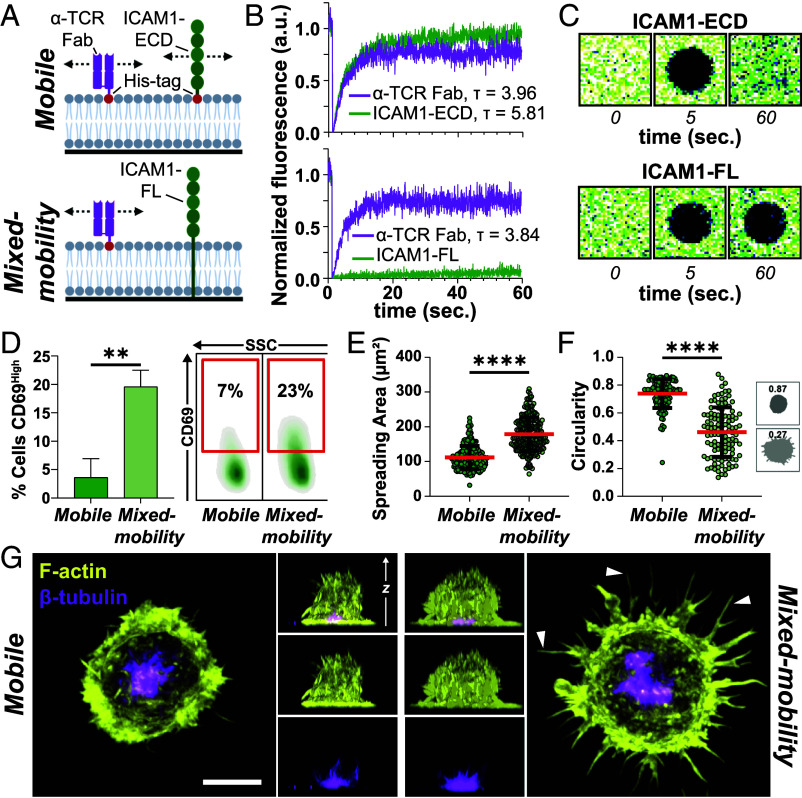
(*A*) Schematic overview over the mobile (*Top*) and mixed-mobility (*Bottom*) SLB system. (*B*) FRAP measurements of fluorescently labeled α-TCR Fab and ICAM1-ECD (*Top*) or α-TCR Fab and ICAM1-FL (*Bottom*). (*C*) Representative example images of fluorescence recovery of ICAM1-ECD (*Top*) and ICAM1-FL (*Bottom*). (*D*) Quantification (*Left*) and representative example (*Right*) of flow cytometry stainings for CD69^High^ T cells after 3 h on mobile and mixed-mobility SLBs and subsequent incubation for 18 h. Unpaired *t* test, **<0.01. Three Biological replicates. (*E*) Quantification of the spreading area in µm^2^ and its circularity (*F*) of T cells spreading on mobile or mixed-mobility SLBs. Green circles represent individual cells. Mann–Whitney test, ****<0.0001. Mean (red lines) ± SD (black bars), three Biological replicates. (*G*) Representative maximum intensity projections and orthogonal views of Airyscan confocal microscopy z-stacks of T cells seeded onto mobile and *mixed-mobility* SLBs, fixed and permeabilized after 15 min. of interaction and stained with Phalloidin (F-actin, yellow) and a directly labeled antibody for β-tubulin (magenta). (Scale bar, 5 µm.) White arrows demarcate filopodial projections on mixed-mobility SLBs.

To establish a reductionist system that enables the simultaneous presentation of laterally mobile and immobile ligands of T cell–expressed receptors, we built on previous findings showing that, in SLBs formed from proteoliposomes containing type I transmembrane (TM) proteins with large extracellular domains (ECDs) and relatively short cytoplasmic tails, the ECD is exposed to the medium while the cytoplasmic domain becomes trapped against the glass surface, rendering these proteins laterally immobile (20–22). We hypothesized that reconstitution of full-length, TM ICAM1 (ICAM1-FL) into nickel-nitrilotriacetic acid (Ni-NTA)-containing liposomes would allow the formation of mixed-mobility SLBs on glass surfaces, presenting spatially integrated immobile, glass-anchored ICAM1 alongside mobile, histidine (His)-tagged ligands, in this case the anti-TCR Fab (Fig. 1*A*). In order to accomplish this, we first purified ICAM1-FL using a previously established immunoaffinity purification procedure (23).

ICAM1-FL was solubilized from human spleen tissue in 1% Tritox X-100 detergent, captured on anti-ICAM1 functionalized Agarose beads that were extensively washed, followed by exchange of the detergent to dialyzable 1% n-octyl-β-D-glucopyranoside. ICAM1 was then eluted at pH 3 followed by neutralization. The isolated protein migrated as a single ~76 kDa band on polyacrylamide gels (*SI Appendix*, Fig. S1*A*), consistent with reported apparent molecular weights ranging from ~76 to 114 kDa depending on glycosylation state (24). Subsequently, ICAM1 was reconstituted into 1,2-dioleoyl-sn-glycero-3-phosphocholine (DOPC) proteoliposomes by dialysis as previously described (25) with the addition that we also included 2 mol% of the Ni^2+^ salt of 1,2-dioleoyl-sn-glycero-3-[(N-(5-amino-1-carboxypentyl) iminodiaceticacid) succinyl] (DOGS-NTA). Liposomes of the same composition, but lacking ICAM1-FL, served as controls. The ICAM1-FL density in each batch of proteoliposome preparation was determined by forming SLBs on glass beads, followed by staining with a fluorescently labeled anti-ICAM1 antibody of known fluorophore-to-protein (F/P) ratio and quantitative flow cytometry. The resulting densities ranged from 50 to 200 molecules/µm^2^. SLBs from proteoliposomes and control liposomes were then formed on glass and further functionalized with His-tagged, Alexa Fluor-647–labeled anti-TCR Fab fragments at a density of 30 molecules/µm^2^. Additionally, SLBs formed from control liposomes were loaded with His-tagged ICAM1-ECD to match the predetermined surface densities of ICAM1-FL in the proteoliposome-derived SLBs. ICAM1-FL and ICAM1-ECD on the SLBs were then labeled with an Alexa Fluor 488–conjugated anti-ICAM1 antibody, and fluorescence recovery after photobleaching (FRAP) experiments were performed to assess their lateral mobility.

In both conditions, His-tagged anti-TCR Fab fragments displayed rapid recovery (τ ~ 4 s for a spot radius of 2 µm), with a mobile fraction of almost 100% (Fig. 1 *B* and *C* and *SI Appendix*, Fig. S1*B*). Similarly, ICAM1-ECD exhibited rapid (τ ~ 6 s) and nearly complete recovery after correction for photobleaching, indicating free lateral mobility, as expected. In contrast, ICAM1-FL displayed slow recovery (τ ≫ 1 m) with >95% immobile over 1 min. (Fig. 1 *B* and *C* and *SI Appendix*, Fig. S1*B*). To further investigate potential effects of immobile ICAM1 on the mobility of anti-TCR Fab, we performed point-scan fluorescence correlation spectroscopy (FCS) measurements. The mean diffusion coefficients of anti-TCR Fab fragments, in the presence of either ICAM1-FL or ICAM1-ECD, were indistinguishable and averaged around 1 µm^2^/s. (*SI Appendix*, Fig. S1*C*), consistent with previous measurements for fully mobile proteins in SLBs. Thus, the presence of immobile ICAM1-FL does not impact the mobility of other proteins that interact with the upper leaflet of the SLB.

Additionally, we performed direct stochastic optical reconstruction microscopy (dSTORM) to characterize the nanoscale organization of ICAM1-ECD and ICAM1-FL in SLBs. Data acquisition was followed by hierarchical density-based clustering (26) and cluster analysis. Nanoscale clusters were identified for both conditions, ICAM1-ECD and ICAM1-FL (*SI Appendix*, Fig. S1*D*), whose formation was potentially mediated by oligomerization of their ECDs (27). No significant differences could be observed in protein cluster density (*SI Appendix*, Fig. S1*E*) or cluster area (*SI Appendix*, Fig. S1*F*). We only detected a very small but statistically significant difference in mean protein cluster circularity, from ~0.76 for ICAM1-ECD to ~0.75 for ICAM1-FL (*SI Appendix*, Fig. S1*G*), indicating a slightly different shape between ICAM1-ECD and ICAM1-FL protein clusters. These findings suggest that T cells would initially encounter a comparable molecular distribution of ICAM1 on either surface.

Taken together, our data demonstrate that reconstituting a mixture of full-length TM proteins and His-tagged ECDs on Ni^2+^-NTA-functionalized SLBs enables the formation of mixed-mobility SLBs. From here on, we refer to the SLB containing ICAM1-FL and His-tagged anti-TCR Fab as the mixed-mobility SLB, and to the control surface containing His-tagged ICAM1-ECD and anti-TCR Fab as the mobile SLB.

Next, we sought to study the functional effects of selective ICAM1 immobilization on T cell activation by mobile TCR ligands. To this end, we introduced CD8^+^ T cells to mobile and mixed-mobility SLBs and allowed them to interact for 3 h. The cells were then removed and incubated for another 18 h, after which surface expression of the T cell activation marker CD69 was assessed, consistent with the notion that T cells require a short period of stimulation to execute functional programs (28). While T cells stimulated on mobile SLBs only exhibited low levels of CD69 upregulation, stimulation on mixed-mobility SLBs resulted in significantly higher CD69 expression, suggesting that immobile ICAM1 is a more active costimulator than laterally mobile ICAM1 (Fig. 1*D*).

In order to begin to understand the underlying cell biological principles of this effect, we first quantified the number of attached cells after 15 min of incubation and subsequent fixation. This revealed a moderate but significant effect on overall cell adhesion with more T cells attaching to mixed-mobility SLBs (*SI Appendix*, Fig. S1*H*). Airy-Scan^®^ confocal microscopy of fixed T cells, labeled with phalloidin to visualize F-actin, revealed a different spreading morphology of T cells on mixed-mobility SLBs, characterized by a larger spreading area and numerous filopodial projections, leading to a decrease in circularity compared to mobile SLBs (Fig. 1 *E*–*G*). Notably, staining for β-tubulin showed centrosome polarization toward the interaction plane, a hallmark of cytotoxic IS formation (29), under both mobile and mixed-mobility conditions (Fig. 1*G*).

**Fig. 2.**
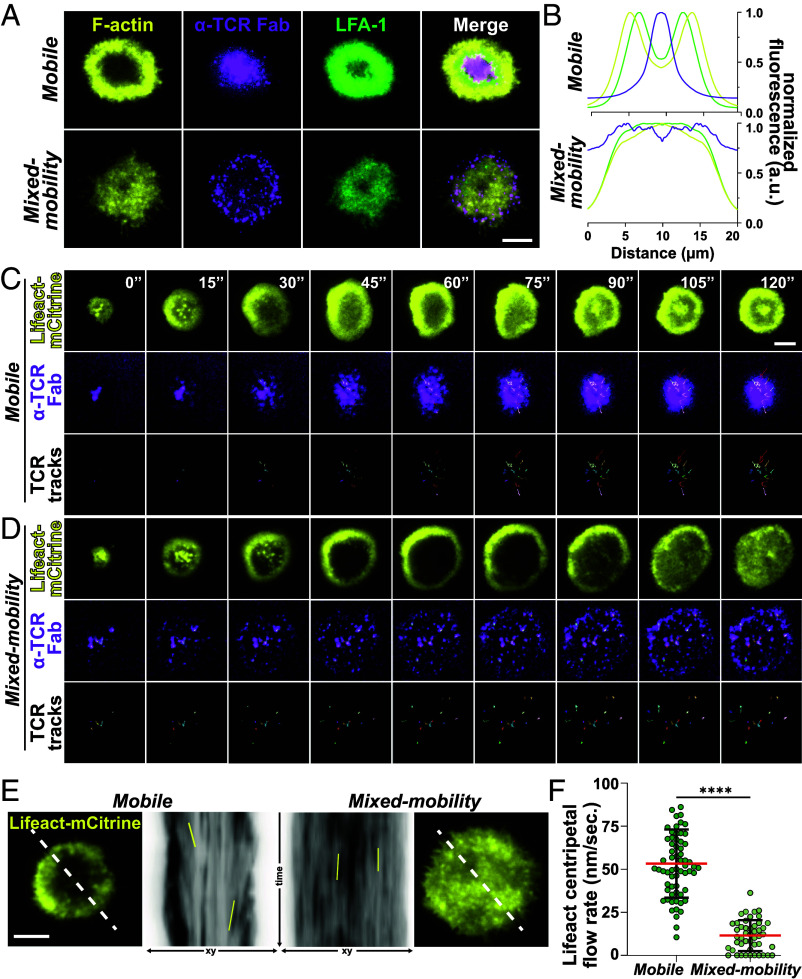
(*A*) Representative TIRF images of synapses formed by T cells on mobile and mixed-mobility SLBs, containing fluorescently labeled α-TCR Fab (magenta) after 15 min. of incubation. Cells were fixed, stained with a directly labeled antibody for LFA-1 (green) and permeabilized and stained with Phalloidin (F-actin, yellow). (Scale bar, 5 µm.) (*B*) Normalized intensity profiles for all labeled proteins in (*A*) across the average intensity projection of radial averages of ≥56 individual synapses formed on mobile or mixed-mobility SLBs synapse in *SI Appendix*, Fig. 2*A*. (*C*) Time-lapse TIRF microscopy of representative examples of Lifeact-mCitrine (yellow, *Top*) transfected T cells interacting with *mobile* or (*D*) mixed-mobility SLBs containing fluorescently labeled α-TCR Fab (magenta, *Middle*). Individual α-TCR Fab tracks are shown in color on black background (*Bottom*). (Scale bar, 5 µm.) (*E*) Representative example images of time-lapse microscopy of Lifeact-mCitrine (yellow) transfected T cells interacting with mobile or mixed-mobility SLBs (outsides). White dashed lines were used for kymograph analysis (*Center*). Solid yellow lines demarcate examples of F-actin features that were used to determine the centripetal flow rate in nm/s. shown in (*F*) unpaired *t* test, ****<0.0001. Mean (red lines) ± SD (black bars). Green circles represent individual measurements from ≥10 individual cells per condition, two Biological replicates.

Next, we used total internal reflection (TIRF) microscopy to assess the organization of the T cell–SLB contact area in more detail. As expected, T cells on mobile SLBs formed canonical synapses with high frequencies, characterized by concentric rings of intense F-actin and LFA-1 and a central accumulation of anti-TCR Fab, the canonical monofocal IS. In stark contrast, T cells on mixed-mobility SLBs primarily formed multifocal ISs in which F-actin and LFA-1 were uniformly distributed throughout the synaptic interface (Fig. 2 *A* and *B* and *SI Appendix*, Fig. S2 *A* and *B*). Strikingly, anti-TCR Fab was not centralized but largely remained in the periphery as multiple discrete, bright microclusters (Fig. 2*A*).

Centripetal flow of F-actin through classic dendritic actin nucleation, cofilin-dependent depolymerization in the distal compartment and myosin-based contractility of antiparallel F-actin bundles drives centralization of TCR microclusters on mobile SLBs (30–32). Additionally, integrin engagement by immobile ligands linked to solid substrates has been shown to slow down F-actin flow in IS formed on anti-TCR coated glass (19, 33). Therefore, we aimed to assess how immobilization of ICAM1 in mixed-mobility SLBs influences F-actin flow and TCR movement. To this end, we introduced Lifeact-mCitrine mRNA into T cells by electroporation, seeded the cells onto SLBs and monitored the first 2 min of IS formation via time-lapse TIRF microscopy. In both SLB types, mobile and mixed-mobility, IS formation began with F-actin foci developing at initial contacts that rapidly expanded through an F-actin rich lamellipodium (Fig. 2 *C* and *D* and Movie S1). In the case of mobile SLBs, initial spreading was accompanied by the appearance of a central region with lower F-actin, a contraction of the overall contact, establishment of the F-actin transport network and the accumulation of TCR microclusters at the center of the IS (Fig. 2 *C*, *E,* and *F*). On the mixed-mobility SLB, the contrast between the initial bright F-actin ring and the interior of the IS was even more pronounced. However, unlike the situation on mobile SLBs, the F-actin signal reappeared in the central region by ~90 s, accompanied by limited contraction, and no uniform centripetal F-actin flow (Fig. 2 *D*–*F* and Movies S1 and S2). Instead, the F-actin network exhibited fluctuations that sometimes appeared to propagate as waves (Movie S2). Notably, while TCR microclusters on mobile SLB exhibited mean flow speeds of ~0.05 µm/s and demonstrated considerable directionality consistent with centripetal flow, they remained relatively static after their initial formation on mixed-mobility SLB, showing significantly reduced speed (*SI Appendix*, Fig. S2*C*) and directionality (*SI Appendix*, Fig. S2 *D* and *E*) compared to mobile SLBs.

These results suggest that, in the mixed-mobility condition, centripetal TCR transport is prevented by the cessation of centripetal actin flow. However, an alternative possibility is that immobilized ICAM1 generates a corralling effect (34) on TCR microclusters that form during cell spreading, whereby immobile TM proteins act as physical obstacles that restrict the lateral movement of neighboring mobile protein clusters, thereby preventing TCR centralization independently of slowed actin flow.

To test this hypothesis, we selected full-length CD44 (CD44-FL) as an unrelated TM protein control, isolated it, and reconstituted it into proteoliposomes. As with ICAM1, CD44-FL was immobilized on the glass surface, whereas the CD44-ECD remained fully mobile (*SI Appendix*, Fig. S3 *A* and *B*). We next added ICAM1-ECD to both CD44-ECD- and CD44-FL-containing SLBs and seeded T cells. Importantly, the presence of immobilized CD44-FL did not prevent TCR centralization or ICAM1 ring formation (*SI Appendix*, Fig. S3 *C* and *D*), indicating that corralling by an unrelated immobile TM protein is not sufficient to explain the observed phenotype.

We further considered whether the effects observed with ICAM1-FL could arise from properties specific to its splenic origin, such as impurities or differences in glycosylation compared with ICAM1-ECD, which we routinely produce in HEK cells. To address this, we engineered a fusion protein comprising the TM and cytoplasmic domains of CD80 fused to an N-terminal SpyCatcher (35) and myc tag (SpyCatcher-TM), enabling the attachment of SpyTag-bearing ligands (*SI Appendix*, Fig. S3*E*). SpyCatcher-TM was expressed in CHO cells, immunoisolated via the myc tag and reconstituted into proteoliposomes. We then generated mixed-mobility SLBs containing either ICAM1-TM or SpyCatcher-TM, alongside mobile control conditions. The latter were loaded with ICAM1-ECD, either carrying a SpyTag sequence or his-tagged ICAM1-ECD, respectively, both produced in HEK cells. Crucially, ICAM1 immobilized via SpyCatcher fully recapitulated the ICAM1-FL mixed-mobility phenotype, including altered synaptic organization and enhanced cell spreading compared with mobile conditions (*SI Appendix*, Fig. S3 *F* and *G*).

Taken together, these data demonstrate that the absence of TCR microcluster centralization under immobilized ICAM1 conditions arises from engagement of immobile ICAM1 by LFA-1, which disrupts the formation of the centripetal F-actin flow required for TCR centralization, independently of ICAM1 source or nonspecific corralling effects. Moreover, the above-described experiments establish that our strategy for selectively immobilizing proteins in SLBs is broadly applicable to a range of TM proteins.

These findings led us to investigate how the multifocal IS might affect TCR downregulation and recycling as these processes have been originally linked to the formation of the cSMAC (10), where a biphasic process occurs: first, TCR-loaded vesicles are released from the plasma membrane, followed by TCR internalization (36). Upon TCR triggering, the receptor is ubiquitinated and recognized by hepatocyte growth factor-regulated tyrosine kinase substrate (HRS), a component of the endosomal sorting complex required for transport. HRS recruits clathrin and mediates the budding of TCR-loaded ectosomes from the plasma membrane. In the second phase, HRS is replaced by the endocytic adaptor protein Epsin-1 (EPN1), which promotes clathrin-dependent endocytosis. While the ectocytic pathway leads to TCR downregulation, EPN1-dependent internalization can either route the TCR for degradation or enable its recycling back to the plasma membrane. This model, originally developed based on data from mobile SLBs, has also been validated in cell–cell synapses (36).

Consistent with this model, T cells interacting with mobile SLBs exhibited increasing recruitment of anti-TCR Fab and high colocalization with HRS at early time points, which gradually declined over the course of the experiment, along with overall HRS levels (*SI Appendix*, Fig. S4 *A*, *C*, *D,* and *F*). In parallel, initially low levels of EPN1 progressively increased, as did its colocalization with anti-TCR Fab, mostly in a central cluster (*SI Appendix*, Fig. S4 *E* and *G*). In contrast, under mixed-mobility conditions, the levels of anti-TCR Fab, HRS, and EPN1 remained relatively stable over time, with significantly lower levels of anti-TCR Fab and HRS compared to mobile SLBs (*SI Appendix*, Fig. S4 *B*–*E*). Colocalization coefficients for HRS/anti-TCR Fab remained comparatively low and unchanged throughout the time course (*SI Appendix*, Fig. S4*F*). In contrast, colocalization between EPN1/anti-TCR Fab, although lower than on mobile SLBs, increased significantly over time (*SI Appendix*, Fig. S4*G*).

**Fig. 3.**
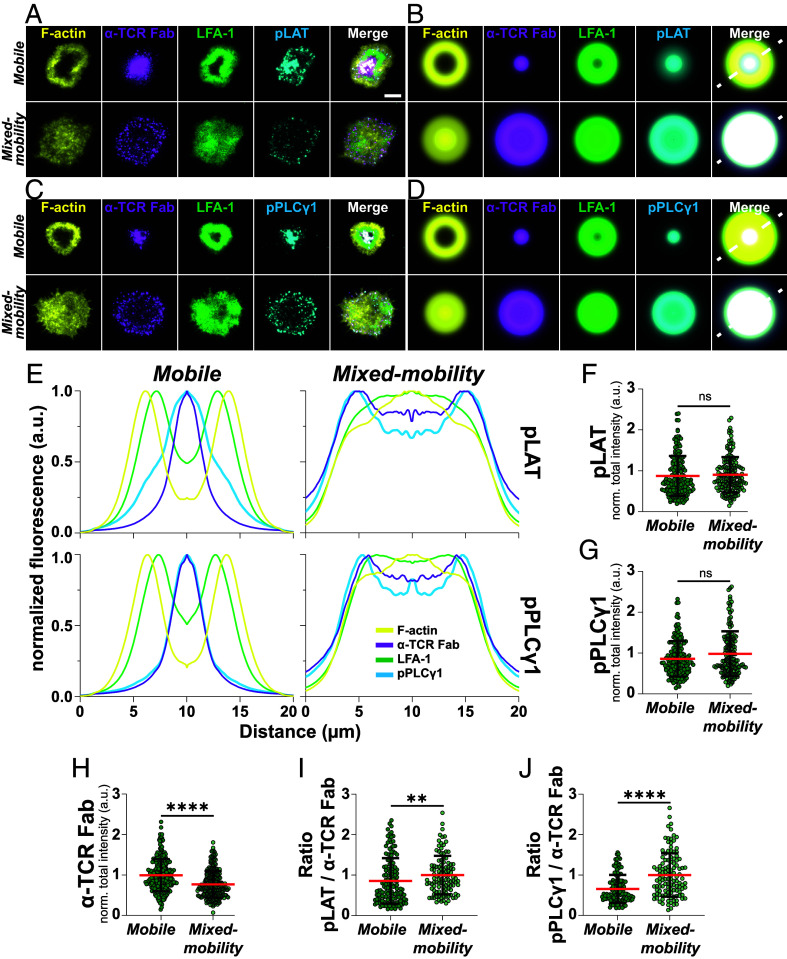
(*A*) Representative TIRF images of synapses formed by T cells on mobile and mixed-mobility SLBs, containing fluorescently labeled α-TCR Fab (magenta) after 15 min. of incubation. Cells were fixed, stained with a directly labeled antibody for LFA-1 (green), permeabilized and stained with Phalloidin (F-actin, yellow) and an antibody for pLAT followed by incubation with fluorescently labeled secondary antibodies. (Scale bar, 5 µm.) (*B*) Average intensity projections of radial averages of ≥45 individual synapses formed on mobile or mixed-mobility SLBs and stained for pLAT. (*C*) Representative TIRF images of synapses formed by T cells on mobile and mixed-mobility SLBs, containing fluorescently labeled α-TCR Fab (magenta) after 15 min. of incubation. Cells were fixed, stained with a directly labeled antibody for LFA-1 (green), permeabilized and stained with Phalloidin (F-actin, yellow) and an antibody for pPLCγ1 followed by incubation with fluorescently labeled secondary antibodies. (*D*) Average intensity projections of radial averages of ≥45 individual synapses formed on mobile or mixed-mobility SLBs and stained for pPLCγ1. (*E*) Normalized intensity profiles along white dashed lines in (*B*)—(*Top*) and (*D*)—(*Bottom*) for all labeled proteins. (*F*) Quantification of normalized pLAT, (*G*) pPLCγ1 and (*H*) α-TCR Fab intensities on mobile and mixed-mobility SLBs. Mann–Whitney test, ****<0.0001. (*I*) Quantification of the ratio between α-TCR Fab and pLAT or pPLCγ1 (*J*) respectively. Mann–Whitney test, **<0.01, ****<0.0001. (*F*–*J*): Mean (red lines) ± SD (black bars). Green circles represent measurements from individual cells. Three Biological replicates.

Next, we assessed how impaired TCR movement and centralization, along with the observed alterations in downregulation and recycling, would affect proximal TCR signaling. To this end, we allowed T cells to interact with SLBs for 15 min, followed by fixation, permeabilization, and antibody staining for the phosphorylated forms of Linker for Activation of T cells (pLAT) and Phospholipase C gamma 1 (pPLCγ1). On mobile SLBs, pLAT localized to both central and more peripheral regions of the IS, while pPLCγ1 was almost exclusively found in the cSMAC alongside anti-TCR Fab (Fig. 3 *A*–*E*). Notably, on mixed-mobility SLBs both proteins underwent a marked shift in localization from central to more peripheral regions of the IS (Fig. 3 *A*–*E*). While overall staining intensities for pLAT and pPLCγ1 across the entire synaptic interface did not significantly differ between conditions, we noted that T cells on mixed-mobility SLBs recruited significantly lower levels of anti-TCR Fab (Fig. 3 *F*–*H*). Interestingly, when pLAT and pPLCγ1 staining intensities were normalized to the level of anti-TCR Fab recruitment in individual cells, we observed significantly increased values for pLAT/TCR and pPLCγ1/TCR under mixed-mobility conditions (Fig. 3 *I* and *J*), suggesting enhanced signaling activity of TCR within microclusters in the multifocal IS.

Taken together, these findings suggest that the multifocal IS dampens TCR downregulation through ectocytosis while maintaining EPN1-mediated recycling. In light of our earlier observation that mixed-mobility SLBs enhance T cell activation (Fig. 1*D*), this increased response may result from impaired TCR turnover and prolonged surface retention of engaged receptors. Sustained presence of signaling-competent TCRs at the plasma membrane could amplify the activation signal despite changes in the spatial organization of the IS. This interpretation aligns with previous findings from SLB systems incorporating physical barriers, where TCRs were trapped in the periphery of the synapse and retained phosphotyrosines for longer than TCRs that translocated to the center of the IS (37). However, then, as here, the effect size was modest.

**Fig. 4.**
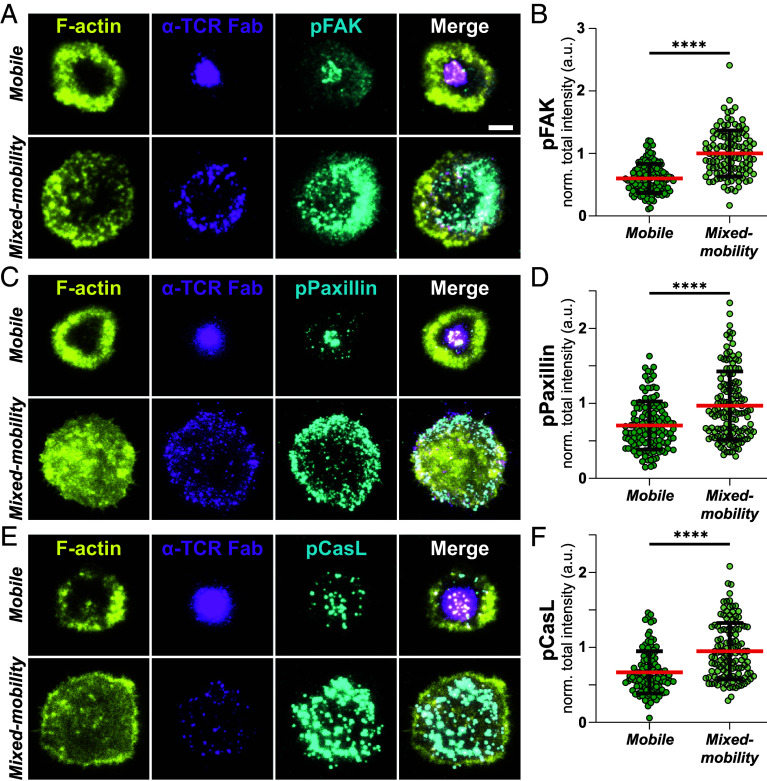
(*A*) Representative TIRF images of synapses formed by T cells on mobile and mixed-mobility SLBs, containing fluorescently labeled α-TCR Fab (magenta) after 15 min. of incubation. Cells were fixed, permeabilized, and stained with Phalloidin (F-actin, yellow) and antibodies for pFAK, pPaxillin (*C*) or pCasL (cyan) (*E*) followed by incubation with fluorescently labeled secondary antibodies. (Scale bar, 5 µm.) (*B*) Quantification of normalized pFAK, pPaxillin (*D*) and pCasL (*F*) intensities on mobile and mixed-mobility SLBs. Mann–Whitney test, ****<0.0001. (*B*, *D,* and *F*): Mean (red lines) ± SD (black bars). Green circles represent measurements from individual cells. Three Biological replicates.

Next, we assessed the impact of selective ICAM1 immobilization on integrin signaling. Upon engagement of LFA-1, Focal Adhesion Kinase (FAK) is recruited to the site of adhesion, where it undergoes autophosphorylation, creating a docking site for additional signaling molecules. One key downstream effector is Paxillin, a scaffold protein that serves as a platform for the assembly of multiprotein signaling complexes, linking integrin engagement to downstream pathways involved in cytoskeletal remodeling and cellular activation (38). To assess integrin activation, we stained T cells interacting with mobile and mixed-mobility SLBs for the phosphorylated forms of these proteins (pPaxillin and pFAK). Importantly, we found that mixed-mobility SLBs induced significantly higher levels of pFAK (Fig. 4 *A* and *B*) and pPaxillin (Fig. 4 *C* and *D*) compared to mobile SLBs. This aligns with earlier findings suggesting that ICAM1, when constrained in its mobility, serves as a more effective ligand for mechanosensitive LFA-1, which requires resistance to the forces exerted by the T cell’s actin cytoskeleton (18, 39). This resistance promotes conformational changes in LFA-1 as well as cytoplasmic adapters like CasL (40) that undergoes a conformational change upon cytoskeletal tension-induced stretching, exposing residues that become phosphorylated (pCasL). In support of this hypothesis, T cells on mixed-mobility SLBs showed significantly stronger staining for pCasL compared to the mobile SLB (Fig.4 *E* and *F*). Notably, these effect sizes were significantly larger than for activating signaling through TCR proximal pathways.

**Fig. 5.**
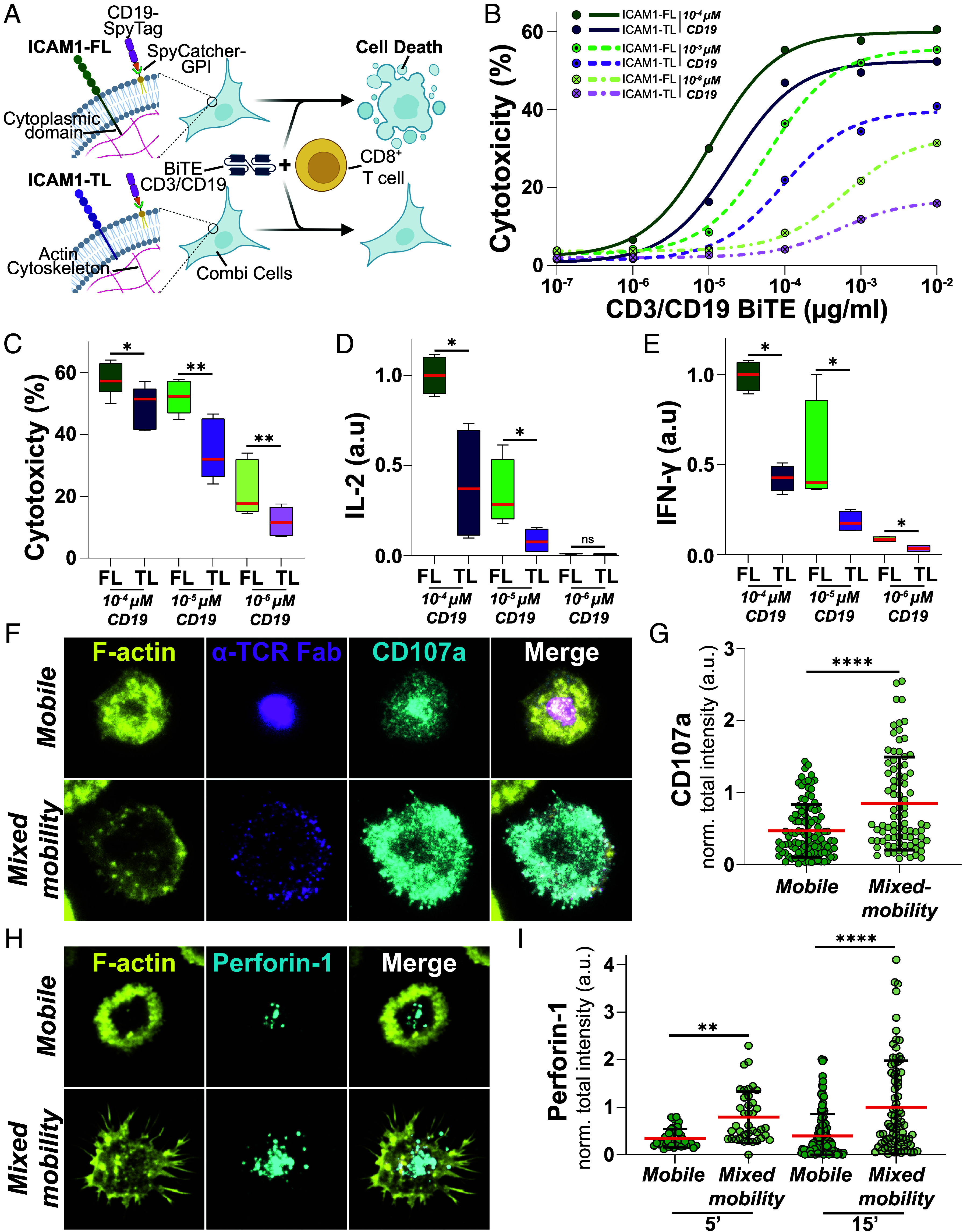
(*A*) Schematic overview over the CombiCell experimental system. (*B*) Mean (circles) and nonlinear fits (lines) of CombiCell cytotoxicity in % expressing ICAM1-FL or ICAM1-TL, respectively loaded with different concentrations of CD19 and in the presence of increasing concentrations of CD3-CD19 BiTEs after 18 h of coincubation in an E:T ratio of 5:1. (*C*) Statistical analysis of cytotoxicity from (*B*) at 10^−2^µg/mL CD3-CD19 BiTE after 18 h of coincubation in an E:T ratio of 5:1 at indicated CD19 concentrations. Paired *t* test, *=0.0165, **≤0.0078. Three Biological replicates with three technical replicates each. (*D*) Normalized quantification of IL-2 and IFN-γ (*E*) levels in the supernatants of CombiCell-T cell cocultures in an E:T ratio of 5:1 in the presence of 10^−2^µg/mL CD3-CD19 BiTE after 24 h. Mann–Whitney test, *=0.0286. Two Biological replicates with three technical replicates each. (*C*–*E*) Box-and-whisker plots show the median (red line) and interquartile range (box), with whiskers indicating minimum and maximum values. (*F*) Representative TIRF images of synapses formed by T cells on mobile and mixed-mobility SLBs, containing fluorescently labeled α-TCR Fab (magenta) in the presence of a directly labeled antibody for CD107a (cyan) after 15 min. of incubation. Cells were fixed, permeabilized, and stained with Phalloidin (F-actin, yellow). (*G*) Quantification of normalized CD107a intensities on mobile and mixed-mobility SLBs. Mann–Whitney test, ****<0.0001. Three biological replicates. (*H*) Representative TIRF images of synapses formed by T cells on mobile and mixed-mobility SLBs, containing fluorescently labeled α-TCR Fab (magenta) after 15 min. of incubation. Cells were fixed, stained with a directly labeled antibody for Perforin-1 (cyan), permeabilized, and stained with Phalloidin (F-actin, yellow). (*I*) Quantification of normalized Perforin-1 intensities on mobile and mixed-mobility SLBs after 5 and 15 min. of incubation. Kruskal–Wallis test, **<0.0001, ****<0.0001. Two (5 min) and three (15 min) biological replicates. (*G* and *I*): Mean (red lines) ± SD (black bars). Green circles represent measurements from individual cells.

We then turned our attention to the effect of ICAM1 immobilization on CD8^+^ T cell activation in a more physiological cell–cell context. To this end, we exploited a newly developed cellular model for immunological studies, termed CombiCells (41). CombiCells are CHO cells in which endogenous ICAM1 has been deleted and that express a GPI-anchored version of SpyCatcher (35), enabling the loading of ligands of choice carrying the SpyTag sequence, in our case CD19. We engineered two derivatives of CombiCells, one expressing a human ICAM1-FL and another expressing a truncated version of TM ICAM1 lacking the cytoplasmic domain (ICAM1-TL) (Fig. 5*A*). We sorted both cell lines to ensure comparable surface expression of ICAM1 (*SI Appendix*, Fig. 5*A*) and confirmed both, the distinct localization of ICAM1 and its confinement in the case of ICAM1-FL, as well as its relative lateral mobility in the case of ICAM1-TL (*SI Appendix*, Fig. 5 *B*–*D*), consistent with earlier studies (18, 42). This setup allowed us to assess the impact of ICAM1-FL and ICAM1-TL on T cell–mediated killing across a wide dynamic range of CD19 densities and concentrations of a CD3/CD19 Bispecific T cell engager (BiTE) (Fig. 5*A*).

ICAM1-FL prompted significantly higher levels of cytotoxicity, as measured by endpoint LDH-release assays, compared to ICAM1-TL across all CD19 loading conditions and a wide range of BiTE concentrations (Fig. 5 *B* and *C*). Additionally, we performed live-cell imaging of T cell–CombiCell cocultures in the presence of fluorescent Annexin-V to monitor real-time killing dynamics. In agreement with our endpoint assays, this confirmed that ICAM1-FL induced significantly higher target cell killing compared to ICAM1-TL, while only minimal cell death occurred under control conditions in the absence of CD3/CD19 BiTEs (*SI Appendix*, Fig. S6 *A* and *B* and Movie S3). Moreover, consistent with our earlier findings in the SLB system, expression of ICAM1-FL in CombiCells resulted in significantly elevated IL-2 and IFN-γ compared to ICAM1-TL, both at individual measurement time points (Fig. 5 *D* and *E*) and when cytokine output was quantified as the integrated response across a 24 h time course (*SI Appendix*, Fig. S6*C*).

To investigate the mechanistic basis of enhanced cytotoxicity in the context of ICAM1-FL, we seeded T cells on mobile and mixed-mobility SLBs in the presence of a fluorescently labeled antibody against CD107a, a marker of lytic granules that becomes exposed on the cell surface upon degranulation. After 20 min of interaction, followed by fixation, we found that CD8^+^ T cells exhibited significantly higher staining intensity on mixed-mobility SLBs compared to mobile SLB conditions (Fig. 5 *F* and *G*). To directly assess cytotoxic effector delivery, we next stained for extracellular Perforin-1, a core component of cytotoxic granules that persists at the IS following release as supramolecular attack particles (43). T cells were incubated on SLBs for 5 or 15 min prior to fixation and staining. While both mobile and mixed-mobility SLBs triggered robust Perforin-1 accumulation at the synapse, mixed-mobility SLBs consistently induced higher total Perforin-1 levels at both time points (Fig. 5 *H* and *I* and *SI Appendix*, Fig. S7 *A* and *B*). At early time points, this increase was primarily associated with larger synaptic contacts and broader Perforin-positive regions under mixed-mobility conditions (*SI Appendix*, Fig. S7 *C* and *D*). In contrast, at 15 min, elevated total Perforin-1 levels were maintained despite a reduction in Perforin-positive area and were instead accompanied by a significant increase in mean synaptic Perforin-1 intensity, consistent with more locally concentrated Perforin accumulation (*SI Appendix*, Fig. S7 *D* and *E*). Together, these findings support a model in which mechanically engaged ICAM1 promotes both enhanced degranulation and sustained, spatially focused delivery of cytotoxic effectors at the IS, in line with recent reports linking lytic granule fusion to mechanically active, high-affinity integrins experiencing pulling forces (44, 45).

Taken together, these data suggest that immobile ICAM1 is significantly more active than mobile ICAM1 at supporting T cell activation and CD8^+^ T cell effector function.

## Discussion

In this study, we introduce a modular, reductionist SLB-based system that enables the simultaneous presentation of laterally mobile and immobile ligands to T cell–expressed receptors. Building upon previous findings that full-length TM proteins are immobile within SLBs, we demonstrate that reconstitution of full-length ICAM1 into Ni^2+^-NTA-functionalized liposomes with additional His-tagged ligands yields SLBs with spatially interspersed mobile and immobile ligands, enabling controlled manipulation of ligand mobility without altering overall surface composition.

Using this system, we demonstrate that selective ICAM1 immobilization enhances CD8^+^ T cell activation and effector functions in response to mobile anti-TCR Fab fragments, indicating that physical anchoring of ICAM1 substantially alters downstream cellular behavior and signaling pathways. These findings are consistent with prior studies using murine DCs and CD4^+^ T cells, which showed that cytoskeletal anchoring of ICAM1 by DCs enhances LFA-1 conformational change, T cell activation, and proliferation (18). Mechanistically, we find that ICAM1 immobilization disrupts centripetal F-actin flow in CD8^+^ T cells. This observation aligns with earlier studies in Jurkat cells demonstrating that engagement of surface-immobilized VCAM-1, together with coadsorbed anti-TCR antibodies, attenuates retrograde actin flow (19, 33). Importantly, while ICAM1 engagement alone does not induce this effect in Jurkat cells, potentially due to their comparatively low expression of LFA-1 (46), the same study showed that ICAM1 engagement is sufficient to slow centripetal actin flow in primary CD4^+^ T cells (19). Our work extends these observations by demonstrating that ICAM1 engagement alone is likewise sufficient to attenuate centripetal F-actin flow in primary CD8^+^ T cells, thereby enhancing their activation, cytokine production, and cytotoxic function.

The key distinction between our approach and previous systems lies in the mobility of the TCR ligand. In many earlier studies, anti-TCR antibodies were surface-immobilized, rendering TCR microclusters immobile regardless of integrin engagement and precluding analysis of microcluster transport. Microcontact-printing approaches using Polydimethylsiloxane (PDMS) stamps have generated *mixed-mobility* conditions by generating micron-scale patterns of immobilized VCAM-1 (19). However, these systems do not recapitulate the nanoscale organization of cell membranes, alter both mobility and clustering of the integrin ligand and did not examine the formation or transport of TCR microclusters. In such settings, dense VCAM-1 clusters might physically impede the lateral movement of TCR microclusters, effectively blocking their centripetal transport. In contrast, in our *mixed-mobility* SLBs, anti-TCR Fab fragments remain freely mobile and capable of clustering with the TCR. Strikingly, while microcluster formation is preserved, their centripetal transport is inhibited, resulting in a multifocal synapse with signaling biased toward the periphery of the IS. Importantly, CD44 control experiments demonstrate that the mere presence of immobilized TM proteins does not inhibit TCR microcluster transport when ICAM1 remains laterally mobile.

These findings connect to the ongoing debate about the role of the cSMAC in TCR signaling. While the cSMAC has historically been viewed as a signaling hub (3, 5), and may facilitate signaling at low peptide concentrations by concentrating engaged receptors (47), multiple lines of evidence suggest it can also serve as a site for signal termination (10, 48, 49). This aligns well with our findings that HRS and the TCR, and later EPN1 and the TCR, are highly colocalized at the center of the IS on mobile SLBs, mediating ectocytosis and, respectively, the internalization of the TCR. Furthermore, although overall staining intensities for pLAT and pPLCγ1 did not differ between mobile and mixed-mobility SLBs, we found that a considerable fraction of both proteins, particularly pPLCγ1, localized to the cSMAC. Given that PLCγ1 has been detected in synaptic ectosomes of CD4^+^ T cells (50), it is tempting to speculate that a substantial fraction of the central pPLCγ1 signal may be present in extracellular vesicles, and thus, would cease to contribute to cellular signaling.

In contrast, under mixed-mobility conditions we find enhanced per-microcluster signaling, as indicated by increased pLAT and pPLCγ1 intensities after normalization for TCR recruitment. This correlates with a shift in TCR trafficking dynamics with a reduction in HRS-mediated ectocytosis while EPN1-dependent TCR internalization is maintained. These findings suggest that the multifocal synapse reduces TCR turnover and prolongs surface retention of signaling competent microclusters, which enhances downstream signaling and increases T cell activation. These findings align with earlier work using SLBs with physical barriers that trap the TCR in the periphery of the IS, generating a small, but significant prolongation of TCR signaling (37). However, it should be noted that in these studies, the transport of both LFA-1/ICAM1 and TCR/pMHC microclusters was blocked, and the effects on integrin signaling were not investigated, an aspect we addressed in this study. We found that, beyond TCR signaling, immobilized ICAM1 enhances integrin signaling. Immobilization promotes stronger recruitment and phosphorylation of FAK and paxillin, in agreement with earlier work showing that mechanical engagement of LFA-1 supports conformational changes in LFA-1 (18, 19, 39).

Taken together, our data support models in which the immobilization of integrin ligands promotes T cell activation by attenuating the cytoskeletal transport and the inactivation of microclusters at the center of the IS, thereby enhancing sustained signaling (33, 51). In addition, integrin signaling itself is enhanced, further contributing to T cell activation. Notably, we observed increased phosphorylation of Paxillin and FAK, indicative of increased mechanotransduction. Elevated phosphorylation of CasL, a stretch-sensitive adaptor protein, further supports the notion that immobilized ICAM1 transduces higher cytoskeletal tension, thereby potentiating mechanosensitive downstream signaling pathways.

We did not observe some of the effects reported in several earlier studies, which likely reflects the specific properties of each experimental system. For example, modulating the mobility of both anti-TCR antibody and ICAM1 by altering the lipid composition of the SLB led to decreased signaling by immobile bilayers (52), but this could be explained by the inability of the TCR to rearrange immobile ligands into optimal microclusters. Similarly, in a system using glass-bound TCR agonist and VCAM-1, TCR signaling was shown to decrease progressively with increasing amounts of VCAM-1, correlating with a reduction in F-actin flow. A smaller, but significant effect was also observed with high concentrations of ICAM1. These findings have been interpreted to suggest that reduced actin flow diminishes mechanical forces on the TCR, thereby impairing signaling (19). However, it is important to note that the density of VCAM-1 or ICAM1 in glass-bound systems is often unclear and may be higher than in the SLB. Excessive integrin engagement under such conditions could increase intermembrane spacing to a point at which the TCR has difficulty accessing its ligands. Notably, this interpretation aligns with observations from transgenic mice with constitutively active LFA-1 (53), or DCs with enhanced coupling of ICAM1 to the cytoskeleton (13), both of which have been associated with dampened T cell activation outside an optimal range. In our system, which reproduces the principal state found in DCs, where a physiological density of ICAM1 is selectively immobilized while the TCR agonist remains mobile (18), we find that centripetal actin flow is substantially diminished, yet the F-actin cytoskeleton remains highly dynamic and may support both integrin arrest of F-actin flow and dynamic TCR engagement.

An alternative, not mutually exclusive, explanation for enhanced T cell activation under mixed-mobility conditions is that lateral mobility of TCR ligands permits continuous diffusion of new ligands into the contact site, allowing ongoing accrual of engaged receptors over time. In this scenario, immobilized integrin ligands provide optimal mechanical stabilization for integrin engagement and efficient mechanotransduction, while mobile TCR ligands maintain a dynamic supply of signaling-competent receptors. By contrast, when TCR ligands are fully immobilized, the available ligand pool is fixed at the time of initial contact, precluding sustained receptor recruitment.

At the level of CD8^+^ effector functions, ICAM1 immobilization leads to increased lytic granule exocytosis and cytotoxicity by CD8^+^ T cells, both in SLB-based assays and in more physiological cocultures. Expression of full-length, immobile ICAM1 in target cells enhances T cell–mediated killing and cytokine secretion across a broad range of BiTE and target antigen concentrations. These findings directly support recent reports that mechanically stabilized integrin engagement enhances degranulation (44, 45)

More broadly, our system represents an important addition to previous approaches aimed at selectively controlling lateral mobility in SLBs. Aforementioned PDMS-stamp-based systems generate protein clusters on the micrometer scale and are ultimately limited by the smallest size of stamp that can be generated (19). In contrast, dSTORM on mixed-mobility SLBs suggests that immobilized proteins are evenly distributed within the SLB, and that protein cluster formation is at the nanometer scale and comparable between mobile controls and immobilized proteins. Another approach, the immobilization of proteins on lithographically nanopatterned surfaces (54), allows for nanometer-scale spacing control but requires specialized equipment and expertise in nanopatterning. By comparison, the mixed-mobility system presented here can be easily implemented by groups already using SLB technologies to investigate how ligand mobility influences cellular behavior. While we focused on ICAM1, we also demonstrated that this system is applicable to other TM proteins and can be readily extended to a wide array of receptors implicated in IS formation, costimulation, or checkpoint inhibition.

Finally, our findings raise intriguing questions in the context of tumor immunology. Tumor cells frequently undergo profound cytoskeletal remodeling (55), which could impact on the lateral mobility of surface proteins, including ligands for T cell–expressed receptors. In this context, drugs targeting the cytoskeleton or its regulatory pathways may inadvertently modulate immune recognition, not by altering ligand expression per se, but by modifying mechanical parameters such as mobility and resistance to cytoskeletal tension. A deeper understanding of these processes could contribute to development and refinement of therapeutic interventions. Furthermore, incorporating targeted mobility constraints into synthetic systems for therapeutic applications, such as artificial APCs (56), may enhance their ability to engage mechanosensitive immune receptors and thereby increase their immunostimulatory potency.

## Material and Methods

### Experimental Systems.

Human CD8^+^ T cells were isolated from peripheral blood of deidentified healthy donors by negative selection and used after short-term activation with anti-CD3/CD28 beads. SLBs were formed on cleaned glass coverslips from DOPC proteoliposomes containing 2 mol% DOGS-NTA(Ni^2+^) and, where indicated, reconstituted full-length TM proteins. Mobile controls were generated by loading His-tagged ECDs onto Ni-NTA SLBs. Mobile anti-TCR Fab was presented as a His-tagged Alexa-647-labeled reagent at defined surface densities. SLBs containing reconstituted TM proteins and His-tagged ligands are referred to as mixed-mobility SLBs. SLBs loaded only with His-tagged ligands are referred to as mobile SLBs.

### Proteins and Reconstitution.

Full-length TM proteins (ICAM1-FL, CD44-FL, SpyCatcher-TM) were affinity purified from cell pellets or deidentified human spleen samples and reconstituted into proteoliposomes by detergent dialysis. Control ECD proteins were produced in-house. Surface densities of reconstituted proteins on SLBs were quantified by fluorescence labeling with antibodies of known F/P ratios and flow cytometry on coated beads. SLBs were assembled to achieve comparable ICAM1 densities (~50 to 200 molecules/µm^2^) across conditions.

### Mobility Measurements.

Lateral mobility of SLB components was assessed by FRAP and FCS. FRAP recovery times and mobile fractions were used to classify ligands as mobile or immobile. Diffusion coefficients for mobile ligands were determined by FCS.

### Superresolution and Nanoscale Analysis.

dSTORM imaging and density-based clustering were used to assess nanoscale organization and cluster statistics of ICAM1-ECD and ICAM1-FL on SLBs.

### Cell-SLB and Cell–Cell Assays.

CD8^+^ T cells were seeded onto SLBs and imaged live or fixed for immunofluorescence with TIRF and Airyscan confocal microscopy. For functional assays, T cells were cocultured with engineered CHO CombiCells expressing either full-length ICAM1 (ICAM1-FL) or a truncated ICAM1 lacking the cytoplasmic domain (ICAM1-TL) and titrated CD3/CD19 BiTEs. Cytotoxicity was assessed by LDH release and live-cell Annexin-V imaging. Cytokines in supernatants were measured by cytometric bead arrays.

### Live Imaging and Analysis.

Time-lapse TIRF microscopy of Lifeact-mCitrine transfected T cells was used to measure F-actin dynamics, centripetal flow rates, and TCR microcluster tracking. Image segmentation, radial averaging, and kymograph analyses were used to quantify spatial distributions, flow velocities, and directionality.

### Endocytic and Ectocytic Markers and Signaling Readouts.

Fixed cells were stained for HRS, EPN1, pLAT, pPLCγ1, pFAK, pPaxillin, and pCasL and analyzed by quantitative fluorescence microscopy. Normalization of phosphorylation signals to local TCR recruitment was performed on a per-cell basis to assess signaling per microcluster.

### Degranulation and Perforin Detection.

Surface CD107a exposure and extracellular Perforin-1 accumulation were detected by fixation of cells after 5 or 20 min. on the SLB and antibody staining, followed by quantitative TIRF imaging.

### Statistics.

The number of biological replicates for each experiment is given in the figure legends. Data are presented as mean ± SD unless otherwise indicated. Statistical tests and significance thresholds are indicated in the figure legends.

## Supplementary Material

Appendix 01 (PDF)

Movie S1. Time-lapse TIRF microscopy of Lifeact-mCitrine (yellow, top) transfected CD8^+^ T cells making first contact with α-TCR Fab (magenta, bottom) containing mobile (left) or *mixed-mobility* (right) SLBs. Frame interval: 3 sec.

Movie S2. Time-lapse TIRF microscopy of Lifeact-mCitrine (yellow) transfected CD8^+^ T cells interacting with mobile (left) or *mixed-mobility* (right) SLBs. Frame interval: 3 sec.

Movie S3. Time-lapse microscopy of CFSE labelled CombiCells (cyan), loaded with 0.0001μM of CD19 and expressing either ICAM1-FL or ICAM1-TL, in co-culture with CD8^+^ T cells in the presence of Annexin-V (magenta), and in the presence or absence of 0.1μg/ml CD3-CD19 BiTE. Frame interval: 15 min.

## Data Availability

All study data are included in the article and/or supporting information.
