# Supplementary material for: Mixed-mobility supported lipid bilayers uncover the role of immobilized ICAM1 on T cell activation and immune synapse organization
Source: Proc Natl Acad Sci U S A. 2026 Mar 10;123(11):e2530126123. doi: 10.1073/pnas.2530126123 (PMC12993941; doi:10.1073/pnas.2530126123)
Supplement: Supplementary file 1 — Appendix 01 (PDF) [file pnas.2530126123.sapp.pdf]

PNAS

Supporting Information for

Mixed-mobility supported lipid bilayers uncover the role of immobilized ICAM1 on T cell activation and immune synapse organization

Alexander Leithner, Audun Kvalvaag, Tanmay Mitra, Salvatore Valvo, Hannah Dada, Ewoud Compeer, Michael I. Barton, Sofia Bustamante Eguiguren, Edward Jenkins, Christoffer Lagerholm, Omer Dushek, Michael L. Dustin

Michael L. Dustin
Email: michael.dustin@kennedy.ox.ac.uk

This PDF file includes:

Material and Methods
Figures S1 to S7
Legends for Movies S1 to S3

SI References

Other supporting materials for this manuscript include the following:

Movies S1 to S3

Material and Methods:

Cell culture: Human primary CD8$^+$ cells and CombiCells were routinely cultured in RPMI (Gibco) medium supplemented with 10% fetal bovine serum, 1% penicillin/streptomycin, 1% L-glutamine, 1% non-essential amino acids and 50 mM HEPES in cell culture treated flasks at 37°C, 5% CO_2 and 100% humidity. CombiCells were split at ~80% confluency by trypsinization. For CD8$^+$ culture and expansion, 50 U/mL recombinant human IL-2 (PeproTech, UK) were added to the culture medium. Primary human CD8$^+$ were isolated from de-identified healthy human donor blood, provided by the National Health Service (NHS) blood service under ethics agreement number 11/H711/7, with commercially available negative selection kits (RosettaSep Human CD8$^+$ T cell Enrichment Cocktail, STEMCELL technologies, UK) following the manufacturers instruction. After isolation, cells were expanded by addition of anti-CD3/anti-CD28 T cell activation beads (Dynabeads ThermoFisher Scientific, UK) at 25 µL/ 10^6 cells for 3 days following the manufacturer's instructions. The magnetic beads were subsequently removed and cells were expanded further at 10^6 cells/mL for an additional 4 days. T cells were either used within 2 days for experiments or cryopreserved.

Lentivirus production and infection: DNA sequences encoding full-length human ICAM1 (ICAM1-FL) and cytoplasmic tail-depleted ICAM1 (ICAM1-TL) were designed based on previously published sequences (1), synthesized and cloned into the pLenti6.3 vector (ThermoFisher). Lentiviruses were produced in HEK293 cells by co-transfecting ICAM1 expression plasmids with the packaging and envelope plasmids pMD2.G (Addgene, #12259) and pΔ8.91 (Addgene, #202687) using the transfection reagent Genejuice (Merck). Supernatants were harvested after 72h, filtered through a 0.45µm filter and either flash frozen or used immediately to infect CombiCells. ICAM1-FL and TL infected CombiCells were expanded for 1 week, after which they were trypsinized, stained for ICAM1 using a fluorescently labelled antibody and sorted for equal surface of expression levels. Sorted ICAM1-FL and TL cells were expanded and frozen in multiple aliquots to perform experiments using cells from low passage numbers. Cultured cells were routinely checked to ensure comparable ICAM1 surface expression levels.

Formation of mobile and mixed-mobility SLBs: The procedure for reconstituting proteins into liposomes was based on previously published protocols (2). ICAM1 and CD44 were isolated from de-identified human spleen tissue. SpyCatcher-TM was stably

expressed in Chinese ovary hamster (CHO) cells by infection with a lentivirus coding for an N-terminal myc-tag, followed by SpyCatcher and the transmembrane and intracellular domains of human CD80. Around 8g of spleen tissue or 5g of cell pellet were homogenized in 50ml lysis buffer (25mM Tris.Cl, 0.15M NaCl, 1% Triton X-100, 5mM EDTA, 5 tablets of Roche protease inhibitor cocktail, pH 8 at 4℃) using a glass homogenizer. The lysate was then filtered through a cell strainer, incubated on ice for 1h and ultracentrifuged at 100.000g for 1h at 4℃. The supernatant was collected, filtered through a 0.2µm filter and either frozen at -80℃ or directly used for the affinity isolation of ICAM1, CD44 or SpyCatcher-TM.

Affinity-isolation matrices for ICAM1, CD44 or SpyCatcher-TM as well as matrices coupled to irrelevant antibodies, were prepared by resuspending 750mg of NHS-Agarose (26197, Thermofisher) with either 25mg of anti-human ICAM1 antibody (BioXcell, BE0020-2-25MG), anti-human CD44 antibody (BioXcell, BE0262-25MG), anti-myc-tag antibody (BioXcell, RT0263-25mg) or IgGs from mouse serum (I5381, Merck) in 10ml of Coupling/Wash buffer (0.1M sodium phosphate, 0.15M NaCl, pH 7.2) followed by end-over-end mixer at 4℃ overnight. Agarose matrices were centrifuged for 1min. at 1000g and washed twice with Coupling/Wash buffer, followed by incubation with Quenching buffer (1M Ethanolamine, pH 7.4) for 15min. and two additional washes with Coupling/Wash buffer. Matrices were either stored in PBS at 4℃ or directly used for the isolation of proteins from tissue lysates.

For affinity isolation, 2.5ml of matrices coupled to irrelevant mouse IgGs or 1.5ml of anti-ICAM1, anti-CD44 or anti-myc functionalized matrices were transferred to 15ml tubes using wide-bore tips. To remove loosely bound antibodies, matrices were washed 3x with low-pH buffer (50mM Glycine, 0.15M NaCl, 1% Triton X-100, pH 3), 3x with high-pH buffer (50mM Triethanolamine, 0.15M NaCl, 1% Triton X-100, pH 12) and 3x with Wash buffer 1 (25mM Tris.Cl, 0.15M NaCl, 1% Triton X-100, pH 8 at 4℃). Lysates were cleared by two sequential incubations with 1ml of irrelevant IgG-coupled agarose matrices for 1h each at 4℃ with end-over-end mixing. Cleared lysates were then incubated overnight at 4℃ with 1.5ml of anti-ICAM1 functionalized agarose matrices, again with end-over-end mixing. Agarose matrices were washed 3x with Wash buffer 1 and 3x with Wash buffer 2 (25mM Tris.Cl, 0.15M NaCl, 1% Octyl-β-glucoside, pH 8 at 4℃) and transferred to a Poly-prep chromatography column (731-1550, Biorad). ICAM1 was eluted by adding 2ml of low-pH elution buffer (50mM Glycine, 0.15M NaCl, 1% Octyl-β-glucoside, pH 3) to the column,

collecting the eluate into 200μl of neutralisation buffer (1M Tris.Cl, 1% Octyl-β-glucoside, pH 8.5). Aliquots of eluate were loaded onto SDS-PAGE for Coomassie staining and Western plotting to confirm protein isolation.

For reconstitution of transmembrane proteins into liposomes, a 20x stock solution containing 2.5% DOGS-NTA(Ni2+) and 97.5% DOPC (all lipid reagents were obtained from Avanti Polar Lipids) at a concentration of 4mM was prepared by mixing appropriate amounts of lipid in chloroform, followed by vacuum desiccation and resuspension into Tris-saline buffer (25mM Tris.Cl, 0.15M NaCl, pH 8 at 4℃) containing 2% Octyl-β-glucoside. The transmembrane protein-containing solution was then mixed with this stock solution to achieve a final 1x concentration. Control liposomes without transmembrane proteins were prepared in the same way, using buffer instead of protein-containing solution. These solutions were transferred to dialysis tubes (GeBaflex maxi, 8kDa MWCO) and dialyzed for 48h against Tris-saline, with 4x buffer exchanges.

For the formation of Glass-supported lipid bilayers, plasma cleaned glass cover slips (SCHOTT UK) were fixed to 6-channel flow chambers (sticky-Slide VI 0.4, Ibidi). Channels were filled with either transmembrane-containing proteoliposome solutions or control liposomes, incubated for 20min., washed twice with flow buffer (20mM HEPES, 1.37mM NaCl, 5mM KCl, 0.5mM Na_2HPO_4, 6mM D-Glucose, 1mM $CaCl_2$, 2mM $MgCl_2$, 1% BSA, pH 7.2) and blocked with 3% BSA in flow buffer for 20min.. This was followed by two additional washes with flow buffer and subsequent incubation with His-tagged, Alexa Fluor 647-labeled α-TCR Fab for both ICAM1-TM and SpyCatcher-TM proteoliposomes. In the case of SpyCatcher-TM proteoliposomes, an additional incubation step with ICAM1-ECD–SpyTag was performed. In both cases, this procedure yielded *mixed-mobility* SLBs presenting immobilized ICAM1. In the case of control and CD44-TM containing liposomes, SLBs were incubated with His-tagged α-TCR Fab and the His-tagged extracellular domain of ICAM1 to obtain *mobile* or CD44-TM *mixed-mobility* SLBs. Specific protein concentrations required to achieve a molecular density of 30 molecules/μm^2 for α-TCR Fab and, for His-tagged ICAM1, to match the ICAM1 density in proteoliposomes, which ranged from ~150 - 300 molecules/μm^2, were determined from flow-cytometry-based calibration experiments on bead-supported lipid bilayers compared to fluorescently-labelled reference beads of known molecular densities (Bangs Laboratories).

FRAP and FCS: For photobleaching experiments, *mobile* and *mixed-mobility* SLBs were stained with an Alexa-488-labelled anti-ICAM1 (HCD54, BioLegend) or anti-CD44 (BJ18, BioLegend) antibody, washed 3x with flow buffer and transferred to a Zeiss LSM980 confocal microscope, equipped with a C-Plan Apo 63x 1.40NA oil immersion objective and using Zeiss 518F23C immersion oil. Before photobleaching, SLBs were imaged for 1sec. at randomly selected regions, bleached and imaged for another 60sec. Adjacent, non-bleached regions served as controls for bleaching corrections. For photobleaching experiments with cells, ICAM1-FL and ICAM1-TL expressing CombiCells were labeled with an Alexa-647-labelled anti-ICAM1 antibody at 4°C for 15 minutes, followed by washing and transfer to the pre-heated microscope stage.

Spot FCS experiments were performed on a Zeiss LSM980 with a Plan Apo 40x 1.20NA water immersion objective and using distilled H2O as immersion liquid. These experiments were performed with a pinhole diameter of 37µm (corresponding to 1 Airy unit at 488 nm) and using internal GaAsP point detectors. These experiments were performed with a laser power setting of 1% of the 488 nm laser and 2% of the 640 nm laser such that we observed no photobleaching during the acquisition.

Data analyses were performed using Fiji ImageJ, FoCuS-point (3) and Graph Pad prism.

dSTORM imaging: The Oxford NanoImager (ONI) microscope was used to generate 2D SMLM images using dSTORM. A 100x objective lens/1.4 NA in oil-immersion was used. 405 nm and 488 nm lasers were used with 2 channels (with dichroic mirror split at 640 nm). Mobile and *mixed-mobility* SLBs were stained with an Alexa-488 labelled anti-ICAM1 antibody as described above. All calibrations and images were performed at 32°C with a supercritical TIRF angle of 51.5° and paired samples acquired with the exact same preparation of dSTORM buffer (320 µl 1x PBS, 40 µl 50% glucose, 40 µl cysteamine 688 (MEA), 4 µl glucose-oxidase). The 488 nm laser was used at 60% laser power to excite anti-ICAM1-Alexa-488. The 405 nm laser was used to promote fluorophore blinking. 8000 frames with an exposure of 30ms were acquired.

The Oxford NanoImager (ONI) analysis platform, CODI, was used to analyse dSTORM images. Constrained clustering algorithm included in CODI was used to identify clusters. Drift correction was applied using the DME algorithm. Frames were filtered first according to acquisition program settings then to exclude the initial excitation peaks for each laser.

The steady state of localizations per frame was included as true blinking localizations. Filtering parameters around the sigma peak were then applied to include a sigma range of ~50-250 nm (Gaussian fit to localize single molecules). Localization precision filtering was then applied to include localization with precision less than or equal to 25 nm. ICAM-1 events were depicted with a display sigma of 5 nm using fixed representation in the representative images, which were downloaded from CODI in TIFF and PNG formats then cropped and converted to PNG micrographs for figures.

Immunofluorescence and live imaging: T cells were added to SLBs in flow buffer at 37 °C with 5% CO_2 for the indicated time periods. Fixation was then performed by adding paraformaldehyde to a final concentration of 4% and incubating for 15min. at 37 °C. For extracellular staining, cells were blocked with 5% BSA in PBS for 1h and incubated with directly labeled primary antibodies (anti-CD11a-AlexaFluor 405 (TS2/4), anti-CD107a-AlexaFluor 647 (H4A3), and anti-Perforin-AlexaFluor 647 (B-D48)) for 1h at a 1:100 dilution. For intracellular stainings, cells were permeabilized with 0.1% Triton X-100 for min., followed by PBS washes and blocking with 5% BSA in PBS for 1h. Directly labeled primary antibodies (anti-β-Tubulin-AlexaFluor 488 (9F3), 1:200; anti-HRS-AlexaFluor 568, Abcam #72053, 1:200; anti-Epsin 1- AlexaFluor 647 (EPR3023), 1:200) or unlabeled primary antibodies (anti-pLAT, Cell Signaling #3581; anti-pPLCγ1, Cell Signaling #2821; anti-pFAK (31H5L17); anti-pPaxillin (E9U9F); anti-pCasL, Cell Signaling #4015) were added at a 1:100 dilution unless otherwise indicated and incubated at 4°C overnight. This was followed by incubation with appropriate fluorescently labeled secondary antibodies and phalloidin to visualize F-actin. For quantification of TIRF images, the F-actin signal was used to train a machine learning-based pixel-classification image segmentation workflow in *ilastik* (4), generating binary synapse masks. These masks were applied in Fiji ImageJ to quantify total fluorescence intensity per synapse in the respective channels following background correction. Statistical analyses were performed using GraphPad Prism.

For confocal microscopy cells were imaged with a Zeiss LSM980 equipped with an AiryScan module and a Plan-Achromat 63X oil immersion objective NA 1.4. TIRF microscopy was performed on an Olympus IX83 microscope equipped with 405, 488, 568 and 640nm laser lines, a 100X oil immersion objective NA 1.45 and an EMCCD camera.

Cell-cell assays: All functional assays involving primary human $CD8^+$ T-cell/CombiCell co-cultures were performed in 96-well transparent flat bottom well plate formats (Costar 96) with a total culture volume of 150 µL and a CombiCell to T cell ratio of 1:5 with a co-incubation time of 18 hours. CombiCells were seeded one day prior to the experiment in 100 µL fully-supplemented culture medium at a concentration of 10.000 cells/well to allow adhesion. On the day of the experiment, CombiCells were loaded with the indicated CD19-SpyTag concentrations for 1h, washed three times with fresh cell culture media, before 50.000 $CD8^+$ T-cells were added for a total volume of 125 µL per well. Dilution series of anti-CD3/CD19 BiTE (Invivogen) were prepared in 25µl per well, resulting in a final co-culture volume of 150 µl per well and the indicated final BiTE concentrations. LDH release assays (CyQuant LDH cytotoxicity assay, Thermo Fischer, USA) and cytokine measurements (BD Cytometric Bead Array (CBA) Human Th1/Th2 Cytokine Cytometric Bead Array Kit) were performed from cell culture supernatants, following the manufacturers instructions.

Live monitoring of cytotoxicity was achieved by staining CombiCells with 20µM CFSE, followed by seeding into the wells of a 18-well slide (iBidi) at 20.000 cells/well and incubation overnight at 37°C, 5% CO_2. CombiCells were loaded with 0.0001µM CD19-SpyTag, washed and T cells were added at 100.000 cells/well in the presence of 0.1µg/ml anti-CD3/CD19 BiTE and 1/200 Red Incucyte Annexin-V dye (Sartorius). Imaging was performed on a LSM980 confocal microscope equipped with an absolute focus module in multi-position mode. Images were collected every 30 min. for 12h. Image analysis was performed by segmenting the CFSE and Annexin-V signals using machine learning based image segmentation software ilastik, followed by calculation of the relative Annexin-V positive area in Fiji ImageJ.

Figures:

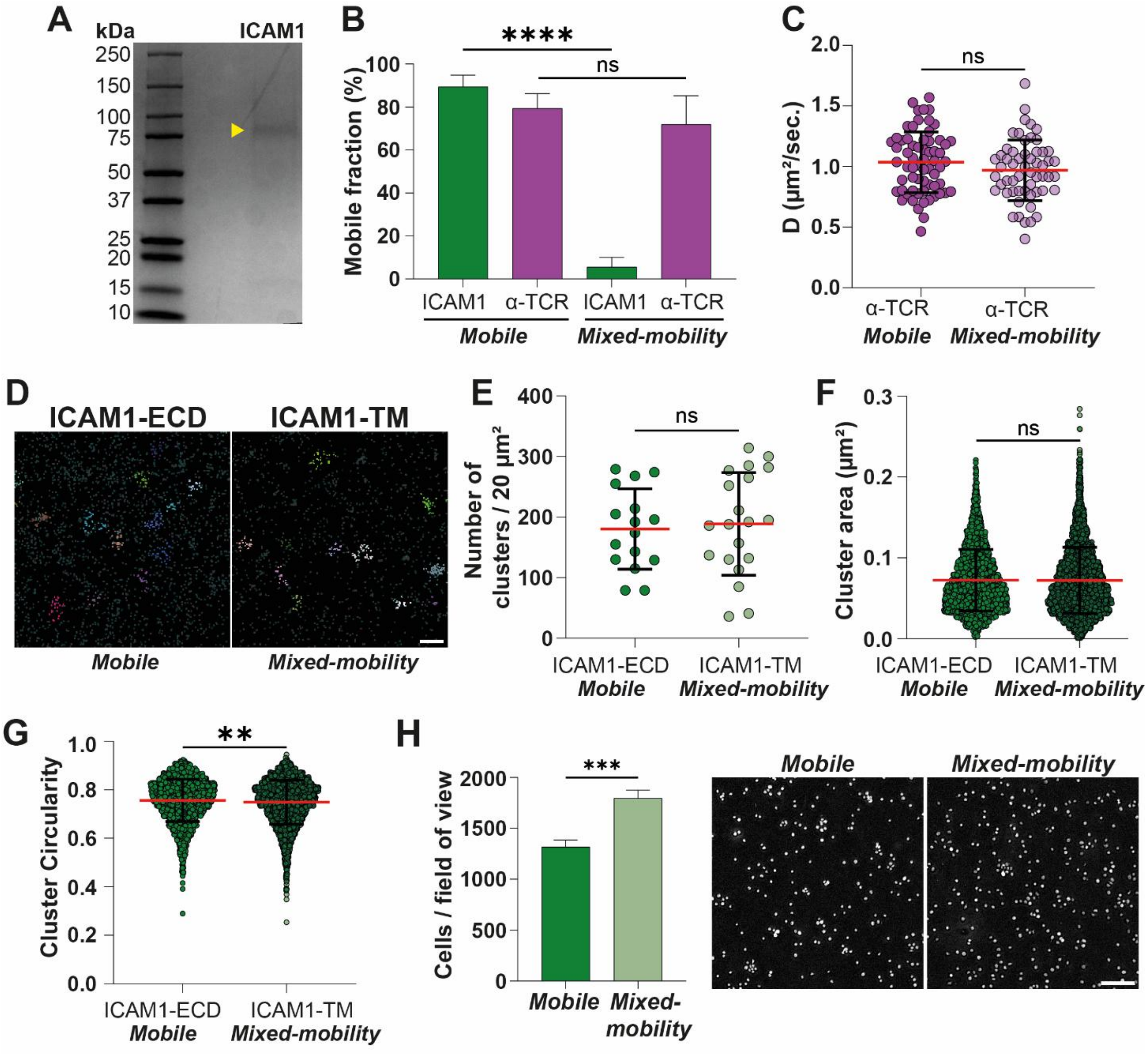

Fig. S1. (**A**) Representative Coomassie-stained polyacrylamide gel of purified splenic ICAM1, indicated by the yellow triangle. (**B**) Quantification of the mobile fraction of ICAM1 and α-TCR Fab in the mobile and *mixed-mobility* SLB. Unpaired t-tests, **** <0.0001. (**C**) Quantification of diffusion coefficients (D) of α-TCR Fab in the context of mobile and *mixed-mobility* SLBs. Unpaired t-test. (**D**) Representative images of ICAM1 clusters identified by dSTORM. (**E**) Quantification of the number of ICAM1-ECD and ICAM1-FL clusters per 20μm^2. Unpaired t-test. (**F**) Quantification of the area of ICAM1-ECD and ICAM1-FL clusters in μm^2. Unpaired t-test. (**G**) Quantification of the circularity of ICAM1-ECD and ICAM1-FL clusters. Unpaired t-test, **<0.01. (B, C, E, F & G): Mean (red lines) ± SD (black bars). (**H**) Quantification (left) and representative example images (right) of cells per field of view on mobile and *mixed-mobility* SLBs after 15min. of incubation. Unpaired t-test, *** = 0.0001. Mean ± SD (black bars). 4 Biological replicates. Scale bar = 20μm.

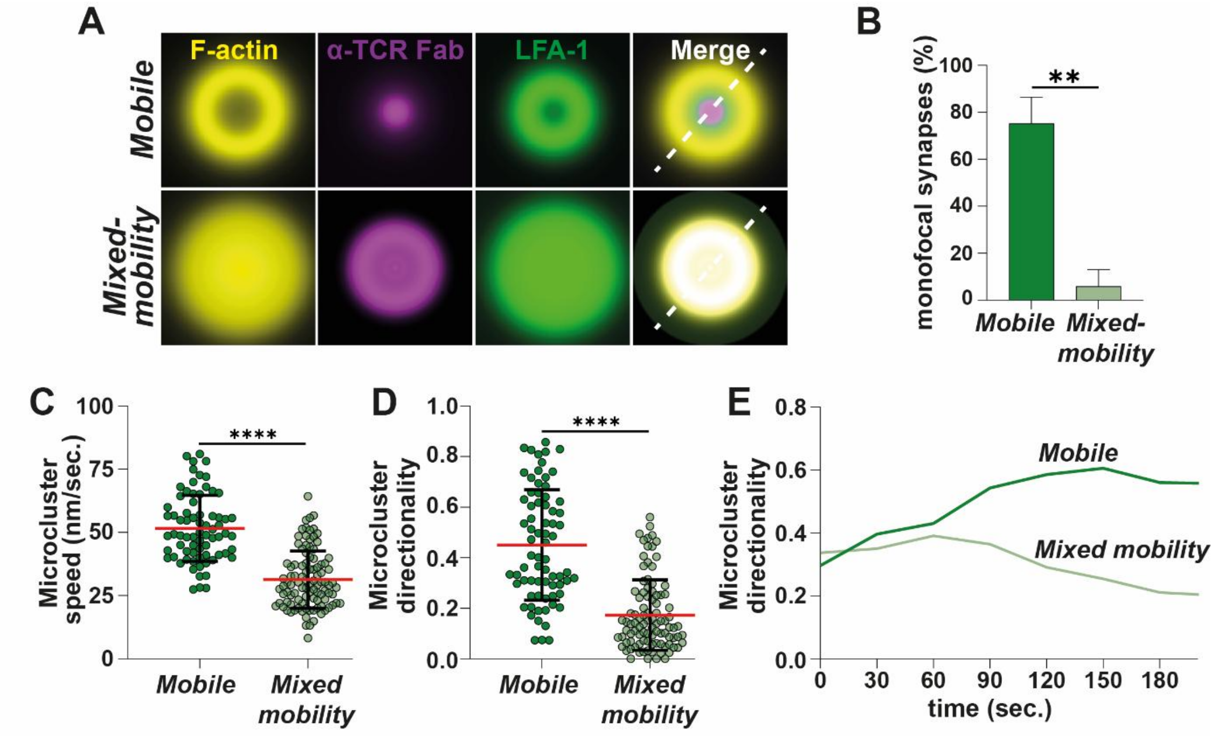

Fig. S2. (**A**) Average intensity projections of radial averages of ≥56 individual synapses formed on *mobile* or *mixed-mobility* SLBs. White dashed lines correspond to intensities plotted in Figure 2B. (**B**) Quantification of monofocal synapses on mobile and *mixed-mobility* SLBs. Mann-Whitney test, ** = 0.0022. Mean ± SD (black bars). 6 Biological replicates. (**C**) Quantification of microcluster speed in nm/sec. and directionality (**D**). Mann-Whitney test, **** < 0.0001. Circles represent individual tracks from 4 different cells per condition. Mean (red lines) ± SD (black bars). 2 Biological replicates. (**E**) Average directionality of microclusters from (D) over time.

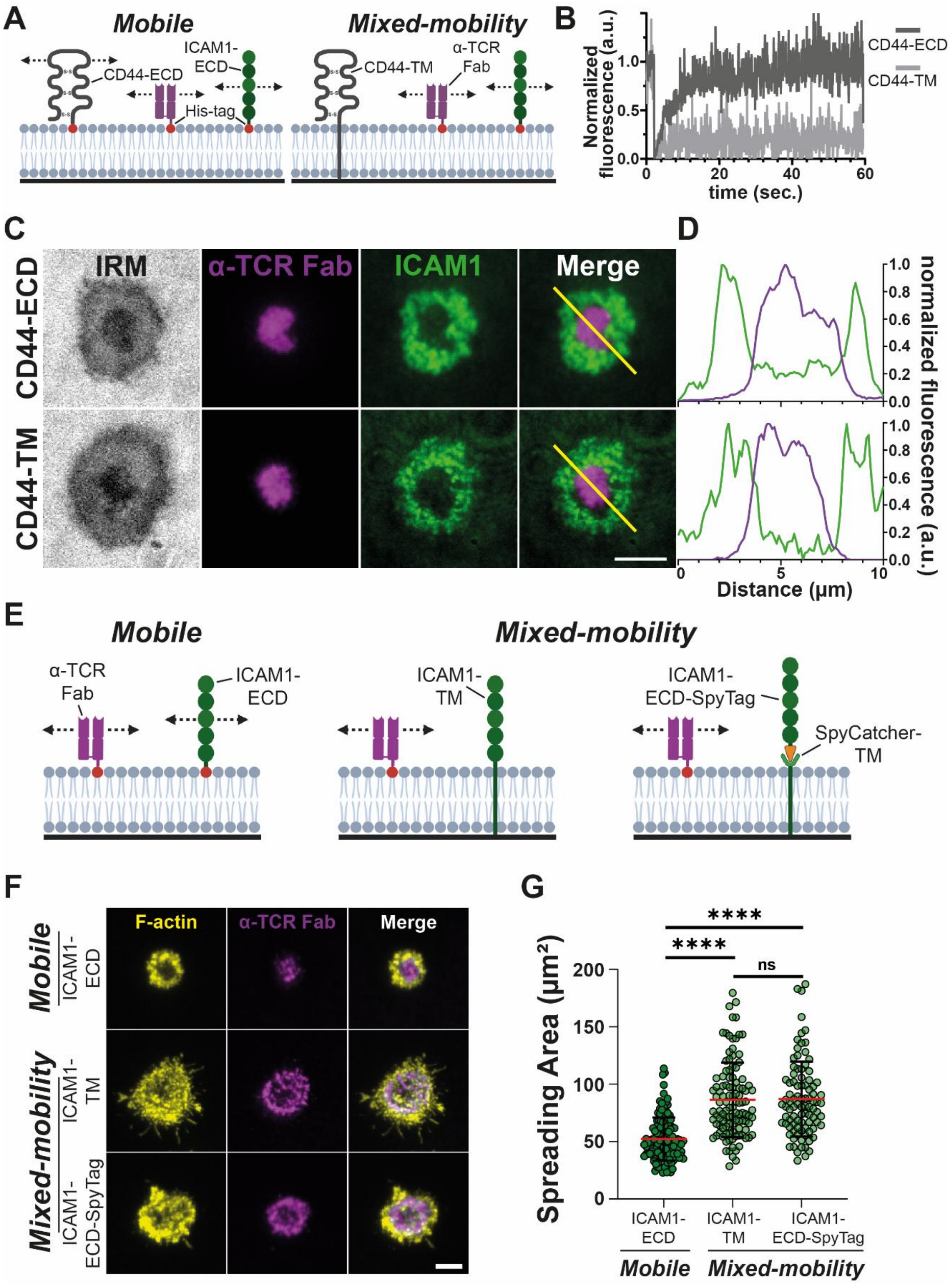

Fig. S3. (**A**) Schematic overview over the *mobile* and *mixed-mobility* SLB system with CD44-ECD (left) and CD44-TM (right) respectively. (**B**) FRAP measurements of fluorescently labelled CD44-ECD and CD44-TM. (**C**) Representative TIRF images of fixed synapses formed by T cells on *mobile* and *mixed-mobility* SLBs, containing either CD44-ECD or CD44-TM, respectively and fluorescently

labelled α-TCR Fab (magenta) and ICAM1-ECD (green) after 15min. of incubation. Scale bar = 5μm. (**D**) Normalized fluorescence intensities of α-TCR Fab (magenta) and ICAM1-ECD (green) along yellow line in (C). (**E**) Schematic overview over the *mobile (*left*)*, *mixed-mobility* with ICAM1-TM (centre) and *mixed-mobility* with SpyCatcher-TM, loaded with ICAM1-ECD-SpyTag, SLB systems. (**F**) Representative TIRF images of synapses formed by T cells on *mobile*, *mixed-mobility* with ICAM1-TM and *mixed-mobility* with SpyCatcher-TM, SLBs loaded with ICAM1-ECD-SpyTag, after 15min. of incubation. Cells were fixed, permeabilized and stained with Phalloidin (F-actin, yellow). Scale bar = 5μm. (**G**) Quantification of the spreading area in μm^2 of T cells spreading on *mobile, mixed-mobility* with ICAM1-TM and *mixed-mobility* with SpyCatcher-TM, loaded with ICAM1-ECD-SpyTag, SLBs. Green circles represent individual cells. Mann-Whitney test, **** < 0.0001. Mean (red lines) ± SD (black bars), 1 Biological replicate.

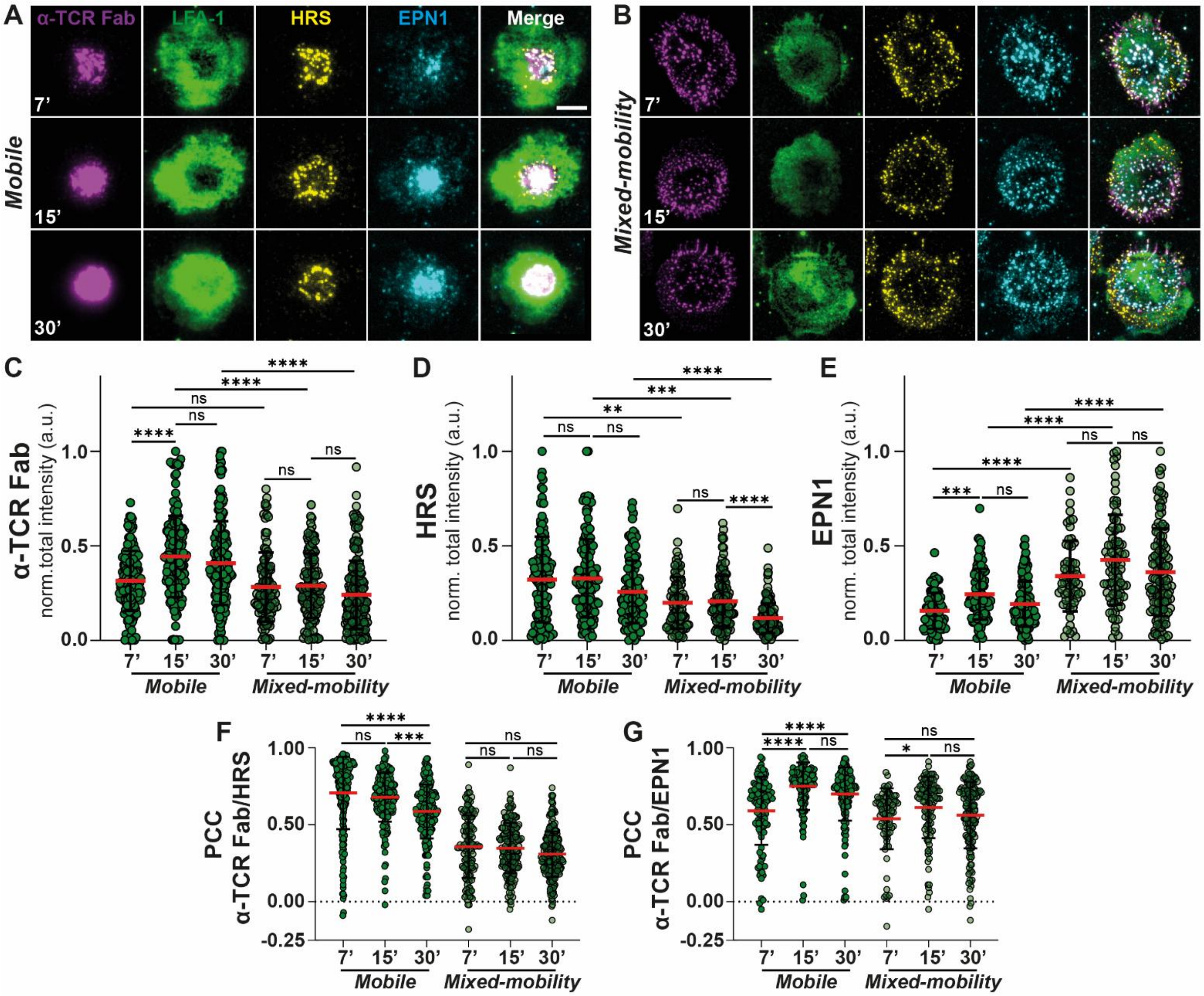

Fig. S4. (**A**) Representative TIRF images of synapses formed by T cells on mobile and *mixed-mobility* SLBs (**B**), containing fluorescently labelled α-TCR Fab (magenta) after 7 (top), 15 (middle) and 30min (bottom). of incubation. Cells were fixed, stained with a directly labelled antibody for LFA-1 (green), permeabilized and stained with directly labelled antibodies for HRS (yellow) and EPN1 (cyan). Scale bar = 5μm. (**C**) Quantification of normalized α-TCR Fab, HRS (**D**) and EPN1 (**E**) intensities after indicated times of incubation on *mobile* and *mixed-mobility* SLBs. Kruskal-Wallis test, ** < 0.01, *** < 0.001, **** < 0.0001. (**F**) Pearson correlation coefficients of α-TCR Fab and HRS or α-TCR Fab and EPN1 (**G**), respectively. Kruskal-Wallis test, * < 0.05, *** < 0.001, **** < 0.0001. (C-G): Green circles represent measurements from individual cells. 3 Biological replicates. Mean (red lines) ± SD (black bars).

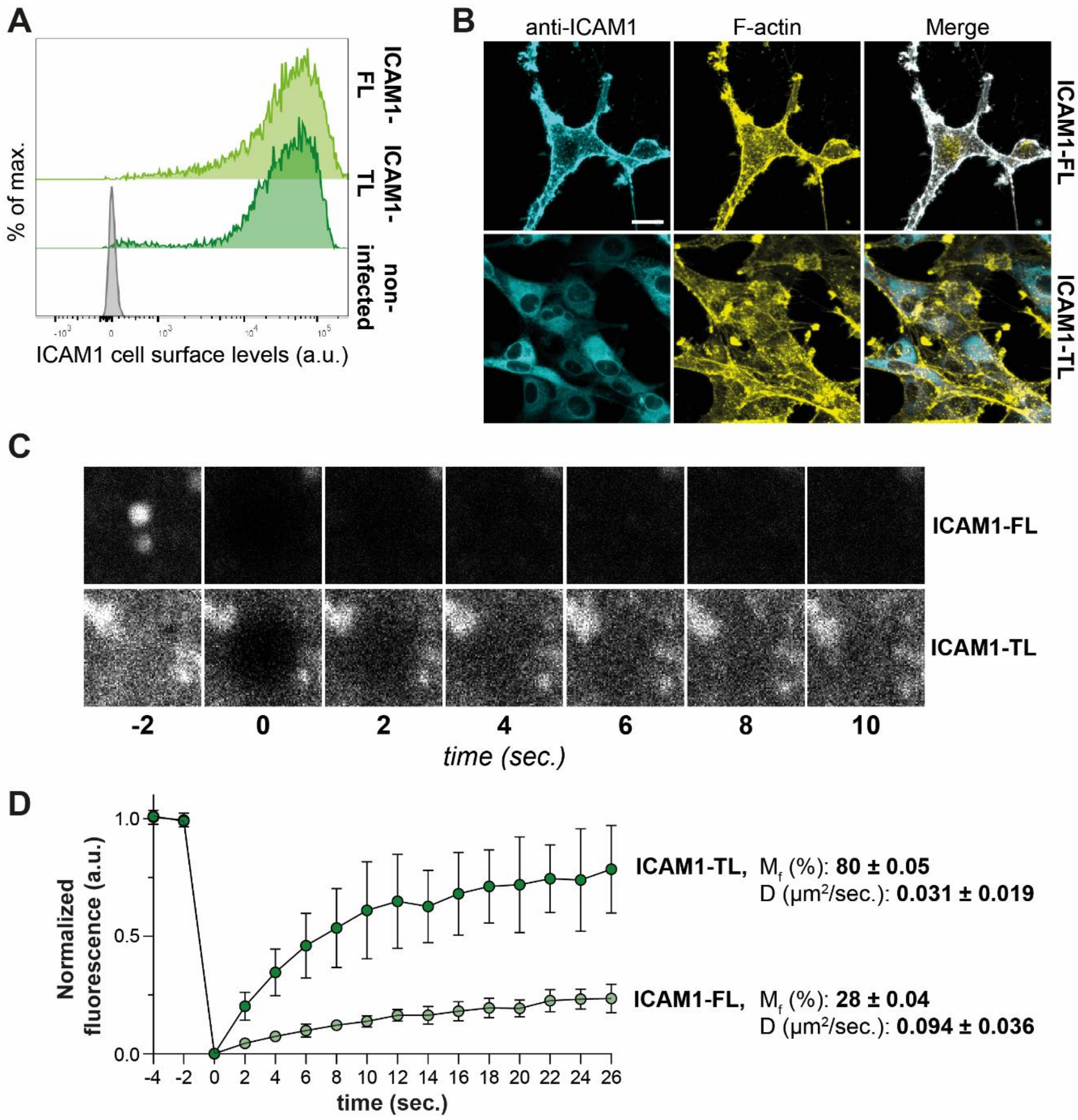

Fig. S5. (**A**) Comparative flow cytometry histograms of sorted CombiCells expressing expressing either ICAM1-FL or ICAM1-TL and surface-stained with a directly labelled antibody for ICAM1. (**B**) Representative maximum intensity projections of ICAM1-FL and ICAM1-TL expressing CombiCells that have been fixed, surface-stained for ICAM1, permeabilized and stained with directly labelled phalloidin. Scale bar = 10µm. (**C**) Representative example of FRAP experiment with ICAM1-FL or ICAM1-TL expressing CombiCells that have been surface-stained with a directly labelled antibody for ICAM1. Timepoint 0 indicated time of photobleaching. (**D**) FRAP measurements of fluorescently labelled ICAM1-FL or ICAM1-TL on CombiCells. Closed circles = Mean, error bars = SD, M_f = mobile fraction, D = diffusion coefficient, n = 6 individual cells per condition from 2 biological replicates.

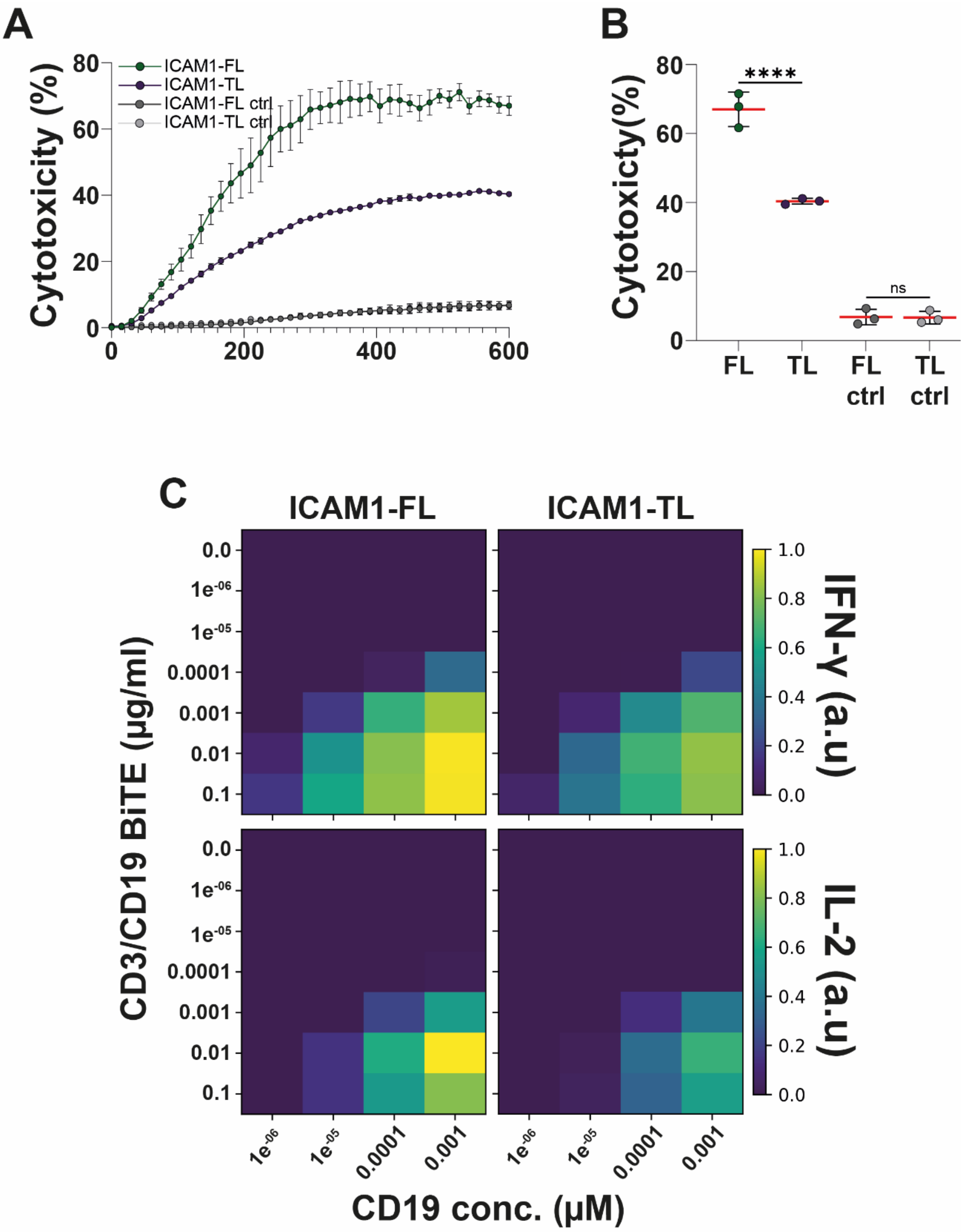

Fig. S6. (**A**) Cytotoxicity over 600min. of ICAM1-FL or ICAM1-TL expressing CombiCells loaded with 0.0001μM of CD19 in co-culture with CD8⁺ T cell in an E:T ratio of 5:1 and in the presence or absence of 0.1μg/ml CD3-CD19 BiTE. Mean (circles) ± SEM (black bars). 3 Biological replicates. (**B**) Quantification of cytotoxicity after 600min. of co-incubation from (A). One-way ANOVA, **** < 0.0001. Mean (red lines) ± SD (black bars). (**C**) Normalized heatmaps of IFN-γ (top) and IL-2 (bottom) in the supernatants of ICAM1-FL (left) and ICAM1-TL (right) expressing CombiCells in co-culture with T cells at an E:T ratio of 5:1 at indicated concentrations of CD3-CD19 BiTE and CD19, quantified as the mean area under the curve (AUC) integrating all measured time points (2, 4, 6, 18 and 24h). 2 Biological replicates with 3 technical replicates each.

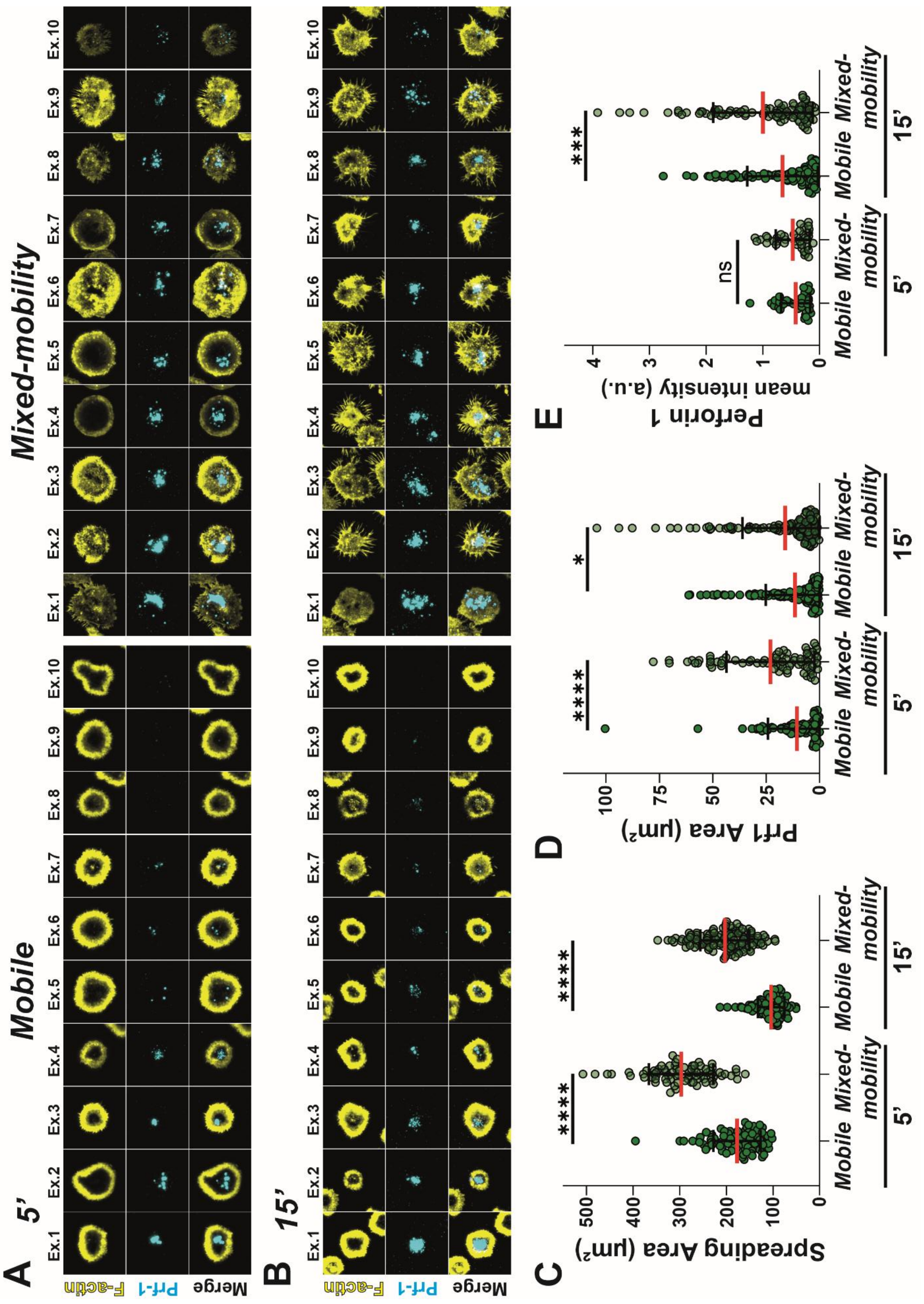

Fig. S7. (**A**) Representative TIRF images of synapses formed by T cells on mobile (left) and *mixed-mobility* (right) SLBs after 5 and (**B**) 15 min. of incubation. Cells were fixed, stained with a directly

labelled antibody for Perforin-1 (cyan), permeabilized and stained with Phalloidin (F-actin, yellow). (**C**) Quantification of the spreading area in μm^2, (**D**) the area occupied by Perforin-1 in μm^2 and (**E**) the mean intensity of Perforin-1 of T cells spreading on *mobile* or *mixed-mobility* SLBs after 5 and 15 min. (C-E): Green circles represent individual cells. Mann-Whitney test, * < 0.05, *** < 0.001, **** < 0.0001. Mean (red lines) ± SD (black bars), 2 (5 min.) and 3 (15 min.) Biological replicates.

Movie S1 (separate file). Time-lapse TIRF microscopy of Lifeact-mCitrine (yellow, top) transfected $CD8^+$ T cells making first contact with α-TCR Fab (magenta, bottom) containing mobile (left) or *mixed-mobility* (right) SLBs. Frame interval: 3 sec.

Movie S2 (separate file). Time-lapse TIRF microscopy of Lifeact-mCitrine (yellow) transfected $CD8^+$ T cells interacting with mobile (left) or *mixed-mobility* (right) SLBs. Frame interval: 3 sec.

Movie S3 (separate file). Time-lapse microscopy of CFSE labelled CombiCells (cyan), loaded with 0.0001µM of CD19 and expressing either ICAM1-FL or ICAM1-TL, in co-culture with $CD8^+$ T cells in the presence of Annexin-V (magenta), and in the presence or absence of 0.1µg/ml CD3-CD19 BiTE. Frame interval: 15 min.

SI References:

1. J. D. van Buul, *et al.*, Inside-out regulation of ICAM-1 dynamics in TNF-alpha-activated endothelium. *PLoS One* **5**, e11336 (2010).
2. M. L. Dustin, T. Starr, R. Varma, V. K. Thomas, Supported planar bilayers for study of the immunological synapse. *Curr Protoc Immunol* **Chapter 18**, 18.13.1-18.13.35 (2007).
3. D. Waithe, M. P. Clausen, E. Sezgin, C. Eggeling, FoCuS-point: software for STED fluorescence correlation and time-gated single photon counting. *Bioinformatics* **32**, 958–960 (2016).
4. S. Berg, *et al.*, Ilastik: Interactive machine learning for (bio)image analysis. *Nat. Methods* **16**, 1226–1232 (2019).
